# Multi-Omics and Single-Cell Dissection Reveals EXT1 as a Glycosylation-Linked Therapeutic Target in Cancer

**DOI:** 10.32604/or.2026.070445

**Published:** 2026-05-21

**Authors:** Wen-Hsin Hsu, Kai-Fu Chang, Chih-Hsuan Chang, Hui-Ru Lin, Chi-Jen Wu, Ching-Chung Ko, Cheng-Chun Wu, Yu-Cheng Ho, Chih-Chun Lin, Chien-Han Yuan, Sachin Kumar, Dahlak Daniel Solomon, Fitria Sari Wulandari, Juan Lorell Ngadio, Do Thi Minh Xuan, Chung-Bao Hsieh, Chung-Chieh Chiao, Ngoc Uyen Nhi Nguyen, Chih-Yang Wang, Yung-Kuo Lee

**Affiliations:** 1 Department of Emergency Medicine, Kaohsiung Armed Forces General Hospital, National Defense Medical University, Kaohsiung, Taiwan; 2 Department of Emergency Medicine, Tri-Service General Hospital, National Defense Medical University, Taipei, Taiwan; 3 Medical Laboratory, Medical Education and Research Center, Kaohsiung Armed Forces General Hospital, National Defense Medical University, Kaohsiung, Taiwan; 4 Division of Experimental Surgery Center, Department of Surgery, Tri-Service General Hospital, National Defense Medical University, Taipei, Taiwan; 5 Institute of Medical Science and Technology, National Sun Yat-Sen University, Kaohsiung, Taiwan; 6 Nursing Department, Kaohsiung Armed Forces General Hospital, National Defense Medical University, Kaohsiung, Taiwan; 7 College of Nursing, Kaohsiung Medical University, Kaohsiung, Taiwan; 8 Department of Medical Imaging, Chi-Mei Medical Center, Tainan, Taiwan; 9 Department of Health and Nutrition, Chia Nan University of Pharmacy and Science, Tainan, Taiwan; 10 School of Medicine, College of Medicine, National Sun Yat-Sen University, Kaohsiung, Taiwan; 11 School of Medicine, College of Medicine, I-Shou University, Kaohsiung, Taiwan; 12 Department of Physical Therapy, I-Shou University, Kaohsiung, Taiwan; 13 Department of Otolaryngology, Kaohsiung Armed Forces General Hospital, National Defense Medical University, Kaohsiung, Taiwan; 14 Department of Otolaryngology, National Defense Medical Center, Taipei, Taiwan; 15 Graduate Institute of Cancer Biology and Drug Discovery, College of Medical Science and Technology, Taipei Medical University, Taipei, Taiwan; 16 Faculty of Applied Sciences and Biotechnology, Shoolini University of Biotechnology and Management Sciences, Himachal Pradesh, India; 17 Department of Bioinformatics, School of Life Sciences, Indonesia International Institute for Life Sciences, Jl. Pulomas Barat Kav 88, Jakarta Timur, Indonesia; 18 Faculty of Pharmacy, Van Lang University, 69/68 Dang Thuy Tram Street, Binh Loi Trung Ward, Ho Chi Minh City, Vietnam; 19 Division of General Surgery, Department of Surgery, Tri-Service General Hospital, Taipei, Taiwan; 20 PhD Program for Cancer Molecular Biology and Drug Discovery, College of Medical Science and Technology, Taipei Medical University, Taipei, Taiwan; 21 Center for Regenerative Medicine, University of South Florida Health Heart Institute, Tampa, FL, USA; 22 Division of Cardiology, Department of Internal Medicine, Morsani School of Medicine, University of South Florida, Tampa, FL, USA

**Keywords:** Exostosin-1, inflammation, glycosylation, machine learning, cancer prognosis, immune evasion, immunotherapy biomarker

## Abstract

**Background:** Glycosylation and inflammation are pivotal in tumor progression, yet the specific glycosyltransferases bridging these processes remain poorly defined. This study investigated Exostosin-1 (EXT1), a key enzyme in heparan sulfate (HS) biosynthesis, as a mechanistic bridge connecting inflammation, stromal remodeling, and immune evasion-driven cancers. **Methods:** We used a multi-omics approach including Least Absolute Shrinkage and Selection Operator (LASSO) Cox regression on The Cancer Genome Atlas (TCGA) pan-cancer cohorts, transcriptomics, survival, single-cell RNA sequencing (scRNA-seq), DNA methylation profiling, pathway enrichment analysis (MetaCore), molecular docking, and immunohistochemistry (IHC) on pancreatic adenocarcinoma (PAAD) and lung adenocarcinoma (LUAD) tissue microarrays. **Results:** EXT1 was identified as one of only two genes overlapping inflammation- and glycosylation-related gene sets, strongly associated with poor overall survival (OS), disease-specific survival (DSS), and progression-free survival (PFS) across cancers, particularly in PAAD and LUAD. Pan-cancer profiling revealed broad EXT1 upregulation. ScRNA-seq localizedEXT1to stromal cells (pancreatic stellate cells, fibroblasts) and epithelial cells co-expressed with immune checkpoint markers. EXT1 promoter hypomethylation correlated with high expression and poor survival. Enrichment analysis of EXT1-correlated genes highlighted activation of critical pro-tumorigenic pathways, including transforming growth factor-β (TGF-β), lysophosphatidic acid (LPA), epidermal growth factor receptor-phosphoinositide 3-kinase-AKT–mitogen-activated protein kinase (EGFR-PI3K-AKT-MAPK), integrin-focal adhesion kinase-Rho GTPase (integrin–FAK–Rho), and epithelial–mesenchymal transition (EMT) pathways. IHC validated stage-dependent EXT1 overexpression in both PAAD and LUAD. **Conclusion:** This multi-omics study identifies EXT1 as a novel link between glycosylation and inflammation. It functions as a driver of stromal activation, immune checkpoint engagement, and tumor progression. EXT1 represents a clinically relevant biomarker and a promising therapeutic target in inflammation-driven cancers like PAAD and LUAD.

## 1 Introduction

Glycosylation and inflammation are fundamental processes that shape cancer biology, but the molecular players bridging these two hallmarks remain incompletely defined [1]. While chronic inflammation drives tumor initiation, angiogenesis, and immune evasion, aberrant glycosylation regulates receptor signaling, cell–cell interactions, and genomic stability. Prior studies have identified the galectin family as a key regulatory hub at this interface, recognizing glycosylation modifications and orchestrating immunosuppressive and pro-inflammatory responses [2]. However, the contribution of glycosyltransferase enzymes directly responsible for glycan biosynthesis remains largely unexplored. In particular, exostosin-1 (EXT1), an enzyme essential for heparan sulfate (HS) biosynthesis, has not been systematically investigated as a mediator of inflammation–glycosylation crosstalk in cancer. Our study addresses this knowledge gap by integrating pan-cancer transcriptomic, epigenomic, and single-cell analyses to dissect the role of EXT1 as a mechanistic bridge linking inflammation, glycosylation, and tumor progression. The inflammatory tumor microenvironment (TME) is characterized by an intricate network of immune cells, cytokines, and stromal components that drive oncogenesis through sustained proliferative signaling, angiogenesis, and suppression of anti-tumor immunity [3,4]. Studies have shown that inflammation can alter gene expression patterns, modulate glycosylation pathways, and influence genomic instability, ultimately shaping tumor progression [5,6]. Despite significant advances in understanding inflammation-driven cancer biology, the key molecular players bridging inflammation, immune modulation, and tumor evolution remain incompletely defined [7]. Notably, previous work has demonstrated that the galectin family functions as a central regulatory hub at the intersection of inflammation and glycosylation [8]. Galectins recognize glycosylation modifications and orchestrate immunosuppression within the TME by promoting T cell apoptosis, enhancing regulatory T cell activity, regulating pro-inflammatory cytokine secretion, and influencing angiogenesis [9,10,11]. While these findings establish galectins as important mediators, other glycosyltransferases such as EXT1 remain poorly characterized in this context. Our study, therefore, addresses this gap by systematically dissecting EXT1 as a potential mechanistic bridge linking inflammation, glycosylation, and tumor progression across cancers.

Glycosylation is an essential post-translational modification that regulates cell-cell communication, immune interactions, and extracellular matrix (ECM) remodeling [12,13]. Aberrant glycosylation has been implicated in tumor metastasis, immune escape, and resistance to therapy, highlighting its crucial role in cancer progression. Recent evidence suggests that inflammatory signaling pathways can directly influence glycosylation patterns, thereby modifying the immunogenicity of tumor cells and affecting immune surveillance. Identifying genes that serve as molecular links between inflammation and glycosylation could provide novel insights into tumor progression and potential therapeutic candidate [14]. With the advent of high-throughput sequencing technologies and large-scale transcriptomic datasets, machine learning (ML) algorithms have become powerful tools for uncovering clinically relevant gene signatures in cancer [15,16]. One such approach is the Least Absolute Shrinkage and Selection Operator (LASSO) regression model, which enables the selection of predictive biomarkers by penalizing overfitting and refining gene selection based on their contribution to survival outcomes [17]. In this study, we employed LASSO Cox regression to develop an inflammation-related gene signature, systematically evaluating its prognostic value across multiple cancer types [18,19,20]. Through pan-cancer analysis, we identified EXT1 as the most significant gene at the intersection of inflammation and glycosylation [21,22]. EXT1 encodes exostosin-1, a glycosyltransferase responsible for HS biosynthesis, a key regulator of cell signaling, tumor-stroma interactions, and immune modulation [23]. Previous studies have linked EXT1 to TME remodeling and immune suppression, yet its role in inflammation-driven cancer progression remains largely unexplored [24].

**Figure 1:**
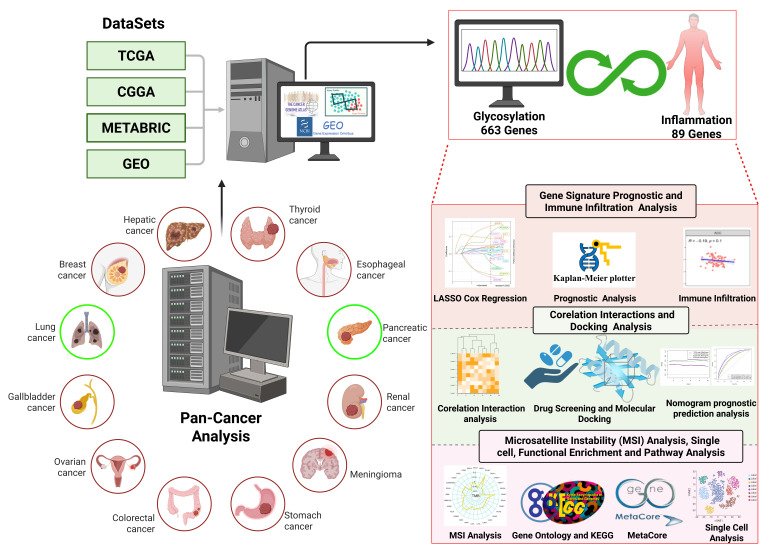
Schematic overview of study design and multi-omics workflow. Publicly available datasets, including TCGA, CGGA, METABRIC, and GEO, were integrated to perform a comprehensive multi-omics analysis across diverse cancer types. Analyses included: (i) development of an inflammation-related gene signature using LASSO Cox regression, Kaplan–Meier survival curves, and immune infiltration profiling; (ii) correlation interaction networks, nomogram prediction modeling, and molecular docking for drug discovery; and (iii) MSI analysis, single-cell transcriptomics, functional enrichment, and MetaCore pathway mapping. This integrative workflow enabled identification of EXT1 as a prognostic biomarker and potential therapeutic target at the intersection of glycosylation, inflammation, and tumor progression.

To comprehensively assess the role of EXT1 in cancer biology, we analyzed its expression patterns, prognostic significance, immune infiltration correlations, and association with genomic instability [25,26]. We further investigated its potential impact on tumor mutation burden (TMB) and microsatellite instability (MSI), two critical biomarkers predictive of response to immune checkpoint inhibitors (ICIs) [27,28]. Given the growing emphasis on precision oncology, understanding the role of EXT1 in immune modulation, inflammation-driven glycosylation, and tumor progression could provide new opportunities for therapeutic intervention [29]. By integrating machine learning-based feature selection, survival modeling, and pan-cancer transcriptomic analysis, this study highlights EXT1 as a central player in inflammation-driven oncogenesis, linking immune suppression, glycosylation, and TME alterations. Our findings suggest that targeting EXT1 may enhance anti-tumor immunity, mitigate inflammation-mediated cancer progression, and improve responses to immunotherapy in selected malignancies (Fig. 1).

## 2 Methods

### 2.1 Selection of Glycosylation- and Inflammation-Related Genes

To investigate the molecular links between inflammation and glycosylation, we curated two gene sets from the Molecular Signatures Database (MSigDB v7.5) [30]. A keyword search identified 89 inflammation-related genes and 663 glycosylation-related genes, which were subsequently analyzed for their functional significance in cancer progression and immune regulation. To construct an inflammation-related prognostic model, we applied a Least Absolute Shrinkage and Selection Operator (LASSO) Cox regression analysis using the ‘glmnet’ package (v4.1-10) in R (v4.4.1). Patients were stratified into high- and low-risk groups based on the median inflammation-related risk score, and prognostic performance was evaluated using Kaplan-Meier survival curves, Cox regression, and Receiver Operating Characteristic (ROC) analyses. Risk score distributions across cancer types were visualized using violin plots generated in R with ‘ggplot2’ (v3.5.1), to illustrate variability in inflammation-related risk scores [31,32,33]. In parallel, we sought to identify genes that simultaneously participate in both inflammatory and glycosylation processes. Venn diagram analysis was performed to compare the inflammation-related gene set (prior to LASSO selection) with the glycosylation-related gene set. This analysis revealed EXT1 and F12 as the only two overlapping candidates. EXT1 was prioritized for downstream analyses because of its strong LASSO regression coefficient, differential expression between tumors and normal tissues, and significant association with survival outcomes [34,35,36].

### 2.2 Construction of the Inflammation-Related Gene Signature

A Least Absolute Shrinkage and Selection Operator (LASSO) Cox regression model was applied to develop an inflammation-related prognostic signature using the ‘glmnet’ package in R (v4.4.1). LASSO regression was chosen for its ability to enhance model interpretability by reducing collinearity and selecting the most predictive genes. The optimal lambda parameter was determined through 10-fold cross-validation, ensuring minimal overfitting. Patients were categorized into high- and low-risk groups based on the median risk score within each dataset.

### 2.3 Prognostic Analysis of the Inflammation-Related Gene Signature

Kaplan-Meier survival analysis was performed to evaluate the prognostic value of the inflammation-related risk score in different cancer types. Survival endpoints, including overall survival (OS), disease-specific survival (DSS), and progression-free survival (PFS), were analyzed using log-rank tests. Univariate and multivariate Cox proportional hazards models were conducted to determine the independent predictive value of the risk score, adjusting for clinical variables such as age, sex, and tumor stage. Hazard ratios (HRs) and 95% confidence intervals (CIs) were visualized using forest plots to illustrate the impact of the signature across cancers. External validation was conducted using Chinese Glioma Genome Atlas (CGGA), Molecular Taxonomy of Breast Cancer International Consortium (METABRIC), and Gene Expression Omnibus (GEO) datasets, all public datasets were loaded and processed using R (version 4.4.1) and the Bioconductor package TCGAbiolinks (version 2.32.0) for data retrieval and manipulation. Predictive performance was assessed via time-dependent ROC curves and concordance index (C-index) calculations [37,38,39].

### 2.4 Identification of EXT1 as a Key Inflammation- and Glycosylation-Related Gene

A Venn diagram analysis was conducted to identify overlapping genes between inflammation- and glycosylation-related gene sets. Among the candidates, EXT1 and F12 were the only two overlapping genes. EXT1 emerged as the most significant gene based on its LASSO regression coefficient, differential expression analysis, and survival impact. The expression of EXT1 was compared between tumor and normal tissues using Wilcoxon rank-sum tests, and its prognostic relevance was confirmed via Kaplan-Meier survival analysis and Cox regression modeling.

### 2.5 Correlation Analysis of EXT1 with Immune Cell Infiltration

To explore the relationship between EXT1 expression and immune infiltration, the ESTIMATE algorithm was used to calculate immune scores, stromal scores, and tumor purity estimates. Additionally, the CIBERSORT deconvolution algorithm was applied to quantify the proportions of 22 immune cell subtypes, including T cells, macrophages, and dendritic cells. Pearson correlation analysis was performed to assess the association between EXT1 expression and immune cell infiltration patterns.

### 2.6 Functional Enrichment Analysis of EXT1-Associated Genes

Differentially expressed genes (DEGs) between high- and low-EXT1 expression groups were identified using ‘DESeq2’ (v1.44.0), applying a cutoff of |log2 fold-change| ≥ 1 and adjusted *p*-value < 0.05. Gene Ontology (GO) and Kyoto Encyclopedia of Genes and Genomes (KEGG) pathway enrichment analyses were performed using the ‘clusterProfiler’ (v4.12.0) package in R (v4.4.1). The functional pathways related to immune response, extracellular matrix remodeling, and glycosylation were prioritized for further analysis.

### 2.7 Association between EXT1 and Genomic Instability (TMB and MSI)

To investigate the relationship between EXT1 expression and genomic instability, the correlation between EXT1 and Tumor Mutation Burden (TMB) and Microsatellite Instability (MSI) was assessed using Spearman’s correlation coefficients. TMB and MSI scores were obtained from The Cancer Genome Atlas (TCGA; accessible via the Genomic Data Commons Data Portal: https://portal.gdc.cancer.gov/) mutation data. These scores were processed, and their correlation with EXT1 expression was visualized using radar plots generated with the ‘fmsb’ R package (version 0.7.6), highlighting cancers where significant associations were observed [38,40].

### 2.8 Nomogram Construction and Validation

A nomogram was developed to improve the clinical applicability of the inflammation-related risk score by integrating EXT1 expression, age, and tumor type to predict 1-, 3-, and 5-year survival probabilities. The predictive performance of the nomogram was assessed using concordance index (C-index) values, calibration curves, and decision curve analysis (DCA).

### 2.9 Single-Cell, Epigenetic, and Pathway Analyses Reveal Context-Specific Regulation of EXT1

Understanding the role of EXT1 within the TME requires resolution beyond bulk datasets, as stromal, immune, and epithelial compartments contribute distinct biological programs. To address this, we applied single-cell RNA sequencing (scRNA-seq) analysis to publicly available datasets from lung and pancreatic cancers, complementing these results with DNA methylation profiling and systems-level pathway enrichment. For LUAD, we analyzed the Human Pancreatic and Lung Cell Atlas published in Science [41], which provides a comprehensive reference of cellular states in pancreatic and lung tissue. Raw data in Hierarchical Data Format (.h5ad) were converted into R-readable format and processed using the ‘Seurat’ package (v5) [42,43]. To determine whether transcriptional dysregulation of EXT1 may also be influenced by epigenetic mechanisms, we integrated DNA methylation analysis using the MethSurv platform [44]. This tool analyzes TCGA Illumina methylome data to quantify CpG methylation in EXT1 and assess its association with patient survival. We specifically interrogated promoter and gene-body CpG sites within EXT1 across LUAD and PAAD cohorts, revealing methylation-dependent regulatory patterns that may underlie tumor-type–specific expression of EXT1. Incorporating methylation profiles into our framework allowed us to connect epigenetic regulation with transcriptional activation at single-cell resolution [45,46,47]. To investigate the downstream signaling programs potentially regulated by EXT1, we performed pathway enrichment analysis using the MetaCore™ v5.4, (Clarivate Analytics) platform, a high-throughput systems biology tool designed to map gene expression data onto curated molecular interaction networks [48,49,50]. EXT1-correlated genes were first identified from TCGA datasets using cBioPortal, where the top 10% of genes showing the highest correlation with EXT1 expression in LUAD and PAAD were extracted [38]. These gene lists were then imported into MetaCore, which applies a comprehensive knowledge base of manually curated pathways to identify statistically enriched biological processes, molecular functions, and regulatory networks. The analysis systematically integrates protein–protein interactions, receptor–ligand relationships, and transcriptional regulation to highlight signaling modules associated with EXT1. This approach provides an unbiased framework to evaluate whether EXT1 expression is linked to recurrent biological themes such as growth factor signaling, ECM remodeling, cytoskeletal regulation, and immune-related pathways, while also enabling comparison of context-specific enrichments across LUAD and PAAD [36].

### 2.10 Drug Screening via Genomics of Drug Sensitivity in Cancer (GDSC), Molecular Docking and Molecular Dynamics

To identify potential therapeutic compounds targeting EXT1, we initially screened drug candidates using correlation analyses from the Genomics of Drug Sensitivity in Cancer (GDSC) and Cancer Therapeutics Response Portal (CTRP) databases via the GSCA platform (https://guolab.wchscu.cn/GSCA/#/) [51]. The top correlated compounds were selected for *in silico* validation. The 3D structure of EXT1 (PDB ID: 7SCH) was retrieved from the RCSB Protein Data Bank (https://www.rcsb.org/) [52]. Protein preprocessing and binding site refinement were carried out using the Protein Preparation Wizard module of Schrödinger (Release Version 2025-4), following standard protocols. The docking grid was centered around the EXT1 binding pocket, specifically near the UDP ligand-binding site, with key amino acid residues Arg346, Lys269, Arg340, and Arg341 defining the active region. Structures of selected drugs were downloaded in SDF format from PubChem (https://pubchem.ncbi.nlm.nih.gov/) and were preprocessed using AutoDock Tools v1.5.6 [53,54]. Molecular docking simulations were performed using AutoDock 4, employing the Lamarckian Genetic Algorithm to evaluate binding interactions at the defined EXT1 active site. The resulting docking poses were visualized and analyzed using Discovery Studio 2024 Client to assess molecular interactions. Subsequently, molecular dynamics (MD) simulations were carried out using GROMACS 2019 on the top-ranked EXT1–ligand complex (based on minimum binding energy from docking) [55]. The protein topology was generated using the Amber FF03 force field via pdb2gmx, while ligand parameters were derived using Gaussian 09 at the B3LYP/6-311G++** level of theory with Grimme’s D3 empirical dispersion correction (GD3BJ). Partial atomic charges were computed via the RESP (Restrained Electrostatic Potential) method. Each protein–ligand complex was embedded in a triclinic box (10 × 10 × 10 Å) and solvated using the TIP3P water model. Neutralization and ionization were achieved with 0.15 M NaCl. Energy minimization was performed at 300 K for 1000 steps, followed by equilibration under NVT and NPT ensembles (100 ps each), using the Nose–Hoover thermostat and Martyna–Tobias–Klein barostat [56,57]. A 100-ns production MD run was conducted with a 2-fs time step under periodic boundary conditions. Key trajectory parameters, including root-mean-square deviation (RMSD), root-mean-square fluctuation (RMSF), radius of gyration (Rg), and hydrogen bonding patterns, were analyzed to assess structural stability and interaction dynamics. The final 10 ns of the trajectory (90–100 ns) were used to calculate binding free energy using the Molecular Mechanics Poisson-Boltzmann Surface Area (MM-PBSA) method [58,59,60].

### 2.11 Clinical Tissue Microarray and Immunohistochemical Analyses

To validate EXT1 protein expression, IHC was performed on formalin-fixed paraffin-embedded (FFPE) clinical tissue microarrays (TMAs). Human LUAD (LC1203a, n = 110 tumors and n = 10 normal lung tissues) and pancreatic adenocarcinoma (PAAD; PA1001e, n = 70 tumors and n = 10 normal pancreatic tissues). TMAs were performed by Hao-Long Biotechnology Ltd. (Kaohsiung City, Taiwan). Informed consent was obtained from all subjects involved in the study.

In addition, the tissues analyzed in this study were derived from previously established tissue microarrays (TMAs) representing pancreatic adenocarcinoma (PAAD) and lung adenocarcinoma (LUAD). PAAD specimens were obtained from the PA1001e TMA (US Biomax Inc., Rockville, MD, USA, https://www.tissuearray.com/tissue-arrays/Pancreas/PA1001e), which includes primary pancreatic ductal adenocarcinoma tissues with corresponding clinicopathological annotations. LUAD specimens were obtained from the LC1501 TMA (US Biomax Inc., https://www.tissuearray.com/tissue-arrays/Lung/LC1501), comprising lung adenocarcinoma tissues across different tumor grades and pathological stages. All tissues were collected by the original commercial providers with prior informed consent from the donors and approval by the appropriate institutional ethics committees, in accordance with the Declaration of Helsinki. No additional patient identifiers were accessible to the authors. Representative immunohistochemical staining patterns from PAAD and LUAD TMAs are shown in the corresponding figure, illustrating inter-tumoral and intra-tumoral heterogeneity across individual cores. Representative tumor and adjacent normal areas were selected by a pathologist from H&E-stained sections, and cylindrical cores (1.5 mm) were arrayed into recipient paraffin blocks. Tissue sections (4 μm) were deparaffinized in pure xylene (two changes, 5 min each), rehydrated through a graded ethanol series (100%, 95%, and 70% ethanol, 3 min each), and subjected to antigen retrieval in 10 mM sodium citrate buffer (pH 6.0). Endogenous peroxidase activity was quenched with 3% H_2_O_2_ for 30 min. Slides were incubated overnight at 4°C with a rabbit polyclonal anti-EXT1 antibody (Thermo Fisher Scientific, cat. no. PA5-112922; Waltham, MA, USA) at a dilution of 1:200. After washing, a biotinylated goat anti-rabbit IgG secondary antibody (Vector Laboratories, Burlingame, CA, USA; cat. no. BA-1000) at 1:500 dilution was applied, followed by visualization using the Vectastain ABC detection system (Vector Laboratories, cat. no. PK-4001) and 3,3′-diaminobenzidine (DAB) chromogen (Vector Laboratories, cat. no. SK-4100). Nuclei were counterstained with commercial hematoxylin solution according to the manufacturer’s instructions.

Adjacent non-tumor tissues from the same tissue microarray were used as internal controls to confirm staining specificity and provide baseline comparison with tumor tissues. All immunohistochemistry procedures were performed by Hao-Long Biotechnology Ltd., (Kaohsiung City, Taiwan). All slides were digitally scanned and analyzed using QuPath v0.3.2. Staining intensity was graded on a four-point scale: 0 = no staining, 1 = weak, 2 = moderate, 3 = strong. All tissues that were successfully stained and imaged were included in the analysis, representing a total of 180 tissue cores from 128 patients. The percentage of positively stained tumor cells was recorded, and a composite H-score was calculated for each specimen using the equation:

H-score = (1 × % weak) + (2 × % moderate) + (3 × % strong),

This method yields a total possible score ranging from 0 to 300. Both staining intensity and percentage positivity were evaluated; however, the H-score was used for statistical analyses and clinical correlations. Stratification was based on clinical stage annotations: pancreatic adenocarcinoma (PAAD) samples were grouped into stages I–IV, and LUAD samples included normal tissues and tumors across stages IA, IB, IIA, IIB, IIIA, IIIB, and IV. Statistical analyses were performed using GraphPad Prism v9.0 (GraphPad Software, San Diego, CA, USA). Group comparisons for LUAD and PAAD were assessed using one-way ANOVA with Tukey’s post hoc test, and glioma samples were analyzed using an unpaired two-tailed *t*-test. Data are reported as mean ± SD.

### 2.12 Patients and Datasets

This study incorporated pan-cancer data from multiple publicly available datasets to ensure a comprehensive and large-scale analysis. The datasets were obtained from four major independent platforms: TCGA (n = 8739), Chinese Glioma Genome Atlas (CGGA, n = 651), Molecular Taxonomy of Breast Cancer International Consortium (METABRIC, n = 1868), and Gene Expression Omnibus (GEO, n = 2459). The TCGA dataset, retrieved from UCSC Xena (https://xenabrowser.net/hub/), covered 32 tumor types while excluding acute myeloid leukemia cases. To ensure rigorous validation, the dataset was randomly split into a training cohort (70%, n = 6117) and a test cohort (30%, n = 2622). The CGGA database, specific to gliomas, included 651 glioma samples, further divided into mRNAseq_693 (n = 422) and mRNAseq_325 (n = 229). The METABRIC dataset, containing 1868 breast cancer patients, was accessed via cBioPortal (https://www.cbioportal.org). Additional datasets from GEO were included to validate findings in non-small cell lung cancer (NSCLC) and breast cancer, incorporating GSE12276, GSE17705, GSE19615, GSE21653, GSE2990, GSE6532_U133A, GSE7390, GSE8894, GSE43767, GSE19188, and GSE67061. These datasets ensured the robustness of the analyses across multiple tumor types and independent patient cohorts.

### 2.13 Statistical Analysis

All statistical analyses were performed using R software (v4.4.1). Survival comparisons were conducted using Kaplan-Meier analysis with log-rank tests. Univariate and multivariate Cox regression models were used to assess the independent predictive value of genes and risk scores. Data visualization, including heatmaps, survival curves, and correlation matrices, was performed using ‘ggplot2’ (v3.5.1), ‘survminer’ (v0.4.9), and ‘pheatmap’ (v1.0.12). Statistical significance was defined as *p* < 0.05.

## 3 Results

### 3.1 Identification of EXT1 within an Inflammation-Associated Prognostic Gene Signature Across Cancers

**Figure 2:**
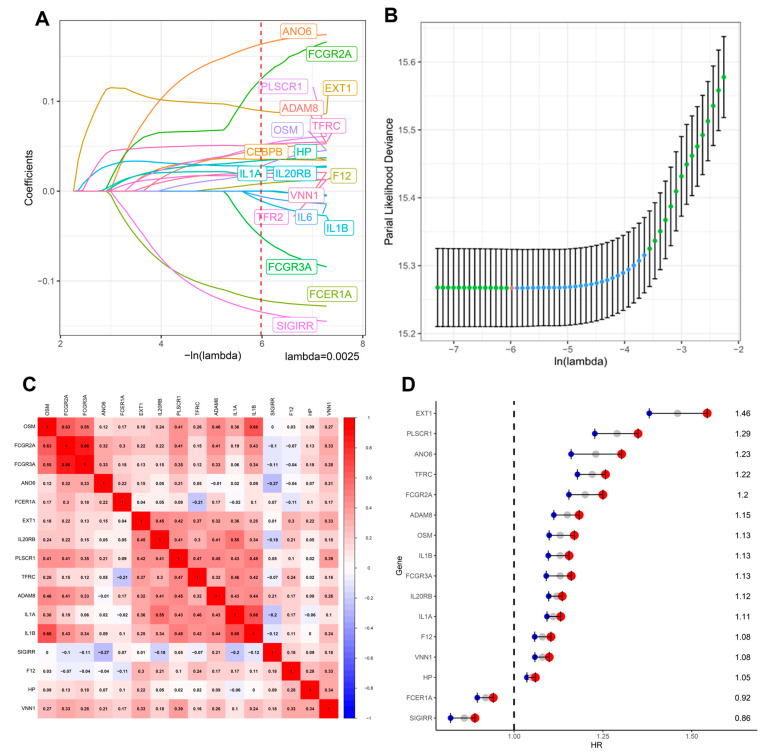
Development of the inflammation-related prognostic gene signature. (**A**) LASSO Cox regression analysis was performed on inflammation-related genes to identify prognostic candidates. Sixteen genes were selected at the optimal λ value (vertical dashed line). (**B**) Cross-validation curve showing the partial likelihood deviance for different λ values, with the minimum λ chosen for model construction. (**C**) Correlation heatmap of the 16 selected genes, illustrating significant co-expression patterns and clustering within inflammatory pathways. (**D**) Univariate Cox regression analysis of the selected genes across pan-cancer datasets. Genes such as EXT1, PLSCR1, and ANO6 were associated with increased HRs > 1, suggesting their potential oncogenic roles, while SIGIRR and FCER1A showed protective effects (HR < 1).

To develop an inflammation-associated prognostic model, we performed LASSO Cox regression on inflammation-related genes curated from MSigDB across pan-cancer datasets. The optimal lambda value (λ = 0.0025) minimized partial likelihood deviance and retained 16 genes with nonzero coefficients (Fig. 2A,B). These genes included EXT1, PLSCR1, ANO6, TFRC, FCGR2A, and others, representing a diverse panel of inflammation-linked regulators. A correlation heatmap (Fig. 2C) revealed distinct co-expression modules, with EXT1 showing moderate to strong correlations (r = 0.32–0.48) with immune-related genes such as OSM and TFRC, suggesting shared functional pathways in immune–glycosylation crosstalk. Univariate Cox regression (Fig. 2D) confirmed the prognostic impact of the selected genes. EXT1 displayed an HR of ~1.46 (95% CI: 1.20–1.78, *p* < 0.01), ranking among the top risk-associated genes in the signature. Similarly, PLSCR1 (HR~1.29) and ANO6 (HR~1.23) were also associated with poor survival. Conversely, genes such as SIGIRR (HR~0.86 and FCER1A (HR~0.92 acted as protective factors. Taken together, this integrative analysis highlights EXT1 as a leading prognostic candidate within the inflammation-related gene signature. Its consistent upregulation, strong HR, and correlation with immune–inflammatory mediators provided the rationale for selecting EXT1 as the focus of subsequent in-depth analyses.

### 3.2 Prognostic Impact of the Inflammation-Related Gene Signature in Training and Test Cohorts

**Figure 3:**
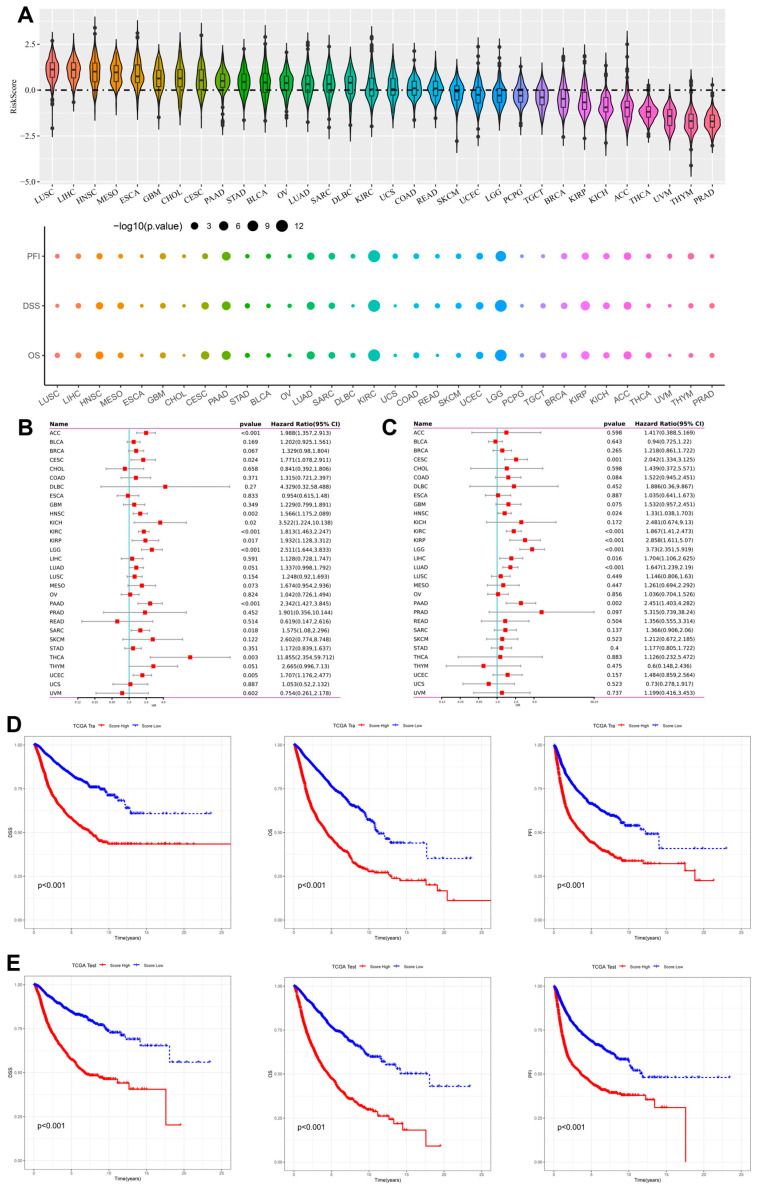
Prognostic impact of the inflammation-related gene signature across cancers. (**A**) Violin plots showing the distribution of risk scores across 33 TCGA cancer types. High-risk scores were enriched in LUSC, LIHC, HNSC, and MESO. Bubble plots (bottom) display the association between risk scores and OS, DSS, and PFI, where bubble size corresponds to the −log10(*p*-value). (**B**) Univariate Cox regression analysis of the inflammation-related risk score for OS across cancer types. Cancers with significant HRs included LUA, PAAD, kidney renal papillary cell carcinoma (KIRP), and lower-grade glioma (LGG). (**C**) Multivariate Cox regression analysis confirmed the risk score as an independent prognostic factor after adjusting for clinical variables. (**D**) Kaplan–Meier survival curves for representative cancers (LUAD, PAAD, KIRC) in the training cohort, showing significantly shorter OS in high-risk groups (*p* < 0.001). (**E**) Kaplan–Meier survival curves in the independent test cohort for the same cancer types (LUAD, PAAD, KIRC), confirming the robustness and reproducibility of the prognostic model (*p* < 0.001).

To evaluate the prognostic relevance of the inflammation-related gene signature, we examined its distribution and association with survival across pan-cancer datasets. The violin plots in Fig. 3A (top) revealed heterogeneous risk score distributions, with elevated scores particularly evident in lung squamous cell carcinoma (LUSC), liver hepatocellular carcinoma (LIHC), head and neck squamous cell carcinoma (HNSC), and mesothelioma (MESO). The bubble plots (Fig. 3A, bottom) showed that higher risk scores were significantly correlated with poor OS, DSS, and PFI across multiple tumor types, with the strongest associations observed in LUAD, colorectal adenocarcinoma (COAD), and kidney renal clear cell carcinoma (KIRC), where *p*-values were consistently < 0.001. Univariate Cox regression (Fig. 3B) confirmed the prognostic significance of the risk score across cancers, with HRs ranging from 1.30 to 2.55 in LUAD, PAAD, KIRP, and LGG, indicating markedly increased risk of mortality in high-risk patients. Multivariate Cox regression (Fig. 3C) demonstrated that the risk score remained an independent predictor even after adjustment for clinical covariates, with LUAD (HR = 1.64 *p* < 0.001) and PAAD (HR = 2.45, *p* = 0.002 showing particularly strong associations. Kaplan–Meier survival analyses further validated these findings. In the training cohort (Fig. 3D), high-risk groups in LUAD, PAAD, KIRC, and LGG exhibited significantly shorter OS (log-rank *p* < 0.001). Importantly, the prognostic effect was reproduced in the independent test cohort (Fig. 3E), where high-risk patients again displayed markedly poorer survival across the same cancer types (log-rank *p* < 0.001). These results highlight the robustness and reproducibility of the inflammation-related gene signature as a prognostic tool across cancers, particularly in LUAD and PAAD, where the risk score consistently stratified patients into high- and low-survival groups. This reinforces the biological and clinical relevance of inflammation-related pathways in driving tumor progression and patient outcomes.

### 3.3 Kaplan-Meier Survival Analysis Validates the Prognostic Value of the Inflammation-Related Gene Signature

**Figure 4:**
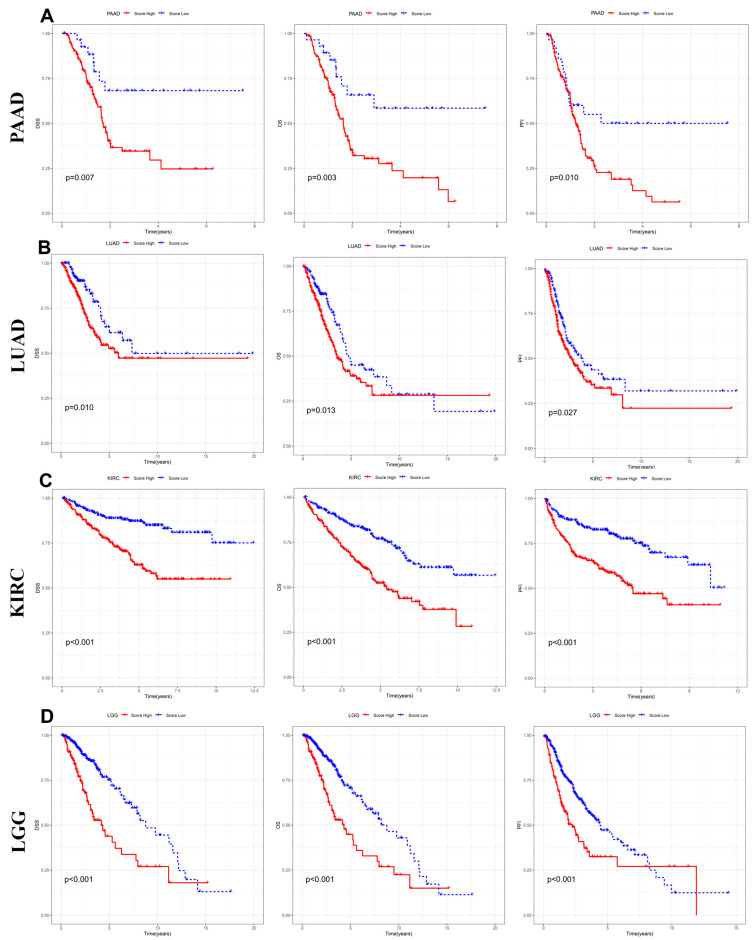
**Kaplan-Meier survival analysis of the inflammation-related gene signature across different cancer types.** Kaplan-Meier survival curves for OS, DSS, and PFI in PAAD (**A**), LUAD (**B**), KIRC (**C**), and lower-grade glioma (LGG) (**D**). Patients were stratified into high- and low-risk groups based on the inflammation-related gene signature.

To assess the prognostic significance of the inflammation-related gene signature, Kaplan-Meier survival analysis was performed on four cancer types, stratifying patients into high- and low-risk groups based on their risk scores. Survival outcomes, including PFI, DSS, and OS, were evaluated. In pancreatic adenocarcinoma (PAAD) (Fig. 4A), patients in the high-risk group exhibited significantly worse OS (*p* = 0.007), DSS (*p* = 0.003), and PFI (*p* = 0.010), compared to those in the low-risk group, indicating that inflammation-related gene expression is associated with disease progression and mortality in PAAD. Similarly, in LUAD (Fig. 4B), high-risk patients had markedly shorter survival times across all three-survival metrics (OS: *p* = 0.10, DSS: *p* = 0.013, and PFI: *p* = 0.027), suggesting that inflammatory processes may contribute to tumor aggressiveness in LUAD. A stronger prognostic association was observed in KIRC (Fig. 4C), where the high-risk group demonstrated significantly poorer PFI, DSS, and OS (all *p* < 0.001), highlighting the inflammation-related signature as a robust predictor of survival in renal cancer. Likewise, in lower-grade glioma (LGG) (Fig. 4D), patients with high-risk scores showed dramatically reduced survival times (PFI, DSS, and OS: all *p* < 0.001), further validating the reliability of the signature in predicting clinical outcomes in brain tumors. These findings confirm that the inflammation-related gene signature is a strong and independent prognostic marker across multiple cancers, with higher risk scores consistently correlating with worse progression-free survival, disease-specific survival, and OS. This signature holds promise for clinical risk stratification and personalized treatment strategies in inflammation-driven malignancies.

### 3.4 Nomogram-Based Prognostic Model Enhances Predictive Performance of the Inflammation-Related Gene Signature

**Figure 5:**
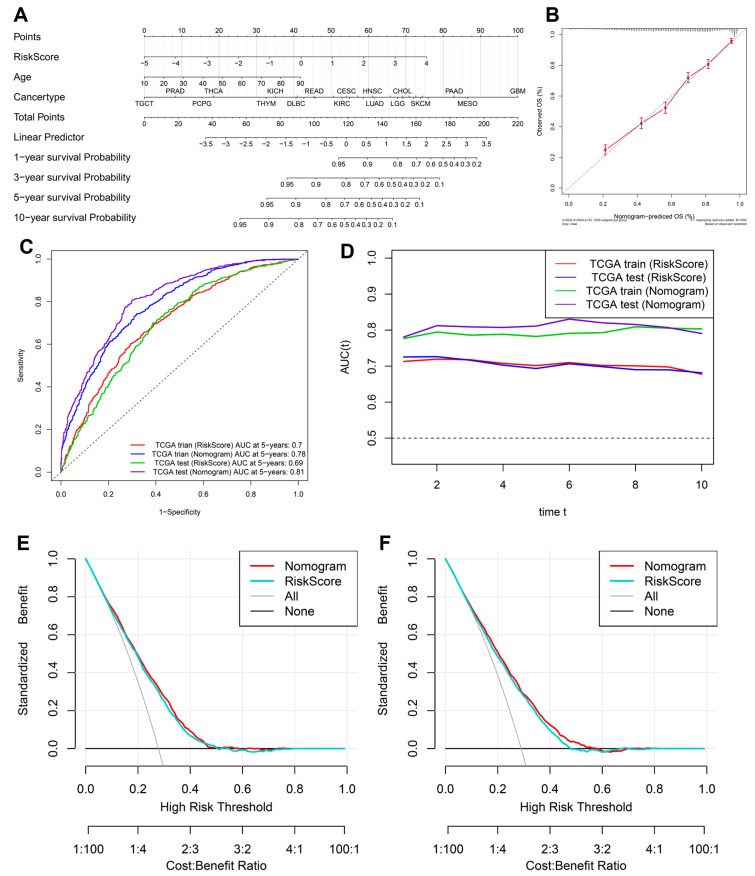
**Construction and validation of a nomogram incorporating the inflammation-related gene signature for prognostic prediction.** (**A**) Nomogram integrating the risk score, age, and cancer type for predicting 1-, 3-, 5-, and 10-year survival probabilities. (**B**) Calibration curve comparing predicted OS from the nomogram with actual OS outcomes. (**C**) ROC curves showing the predictive performance of the risk score and nomogram in the training and test cohorts. (**D**) Time-dependent AUC analysis comparing the prognostic accuracy of the risk score and nomogram. (**E**) and test cohorts (**F**), showing net benefit across risk thresholds.

To improve the clinical applicability of the inflammation-related gene signature, we constructed a nomogram integrating the risk score, age, and cancer type to predict 1-, 3-, 5-, and 10-year survival probabilities (Fig. 5A). The calibration curve (Fig. 5B) demonstrated strong agreement between the nomogram-predicted survival probabilities and actual survival outcomes, indicating its reliability for individualized prognosis estimation. To assess predictive accuracy, we performed ROC analysis in both the training and test cohorts (Fig. 5C). The area under the curve (AUC) values for the 5-year OS prediction were 0.70 (training cohort, risk score), 0.78 (training cohort, nomogram), 0.69 (test cohort, risk score), and 0.81 (test cohort, nomogram). These results indicate that incorporating clinical parameters into the nomogram significantly improves predictive performance over the risk score alone. Time-dependent AUC analysis (Fig. 5D) further confirmed that the nomogram consistently outperformed the risk score in predicting long-term survival across multiple time points, demonstrating its robustness as a prognostic tool. Finally, decision curve analysis (DCA) (Fig. 5E,F) revealed that the nomogram provided superior net clinical benefit compared to the risk score alone in both the training E and test F cohorts, supporting its potential utility for guiding personalized risk assessment and clinical decision-making. Overall, these findings highlight the nomogram as a superior prognostic model, integrating inflammation-related molecular signatures with clinical factors to improve patient stratification and survival prediction across diverse cancers.

### 3.5 Association Between the Inflammation-Related Gene Signature and Malignant Tumor Phenotypes

**Figure 6:**
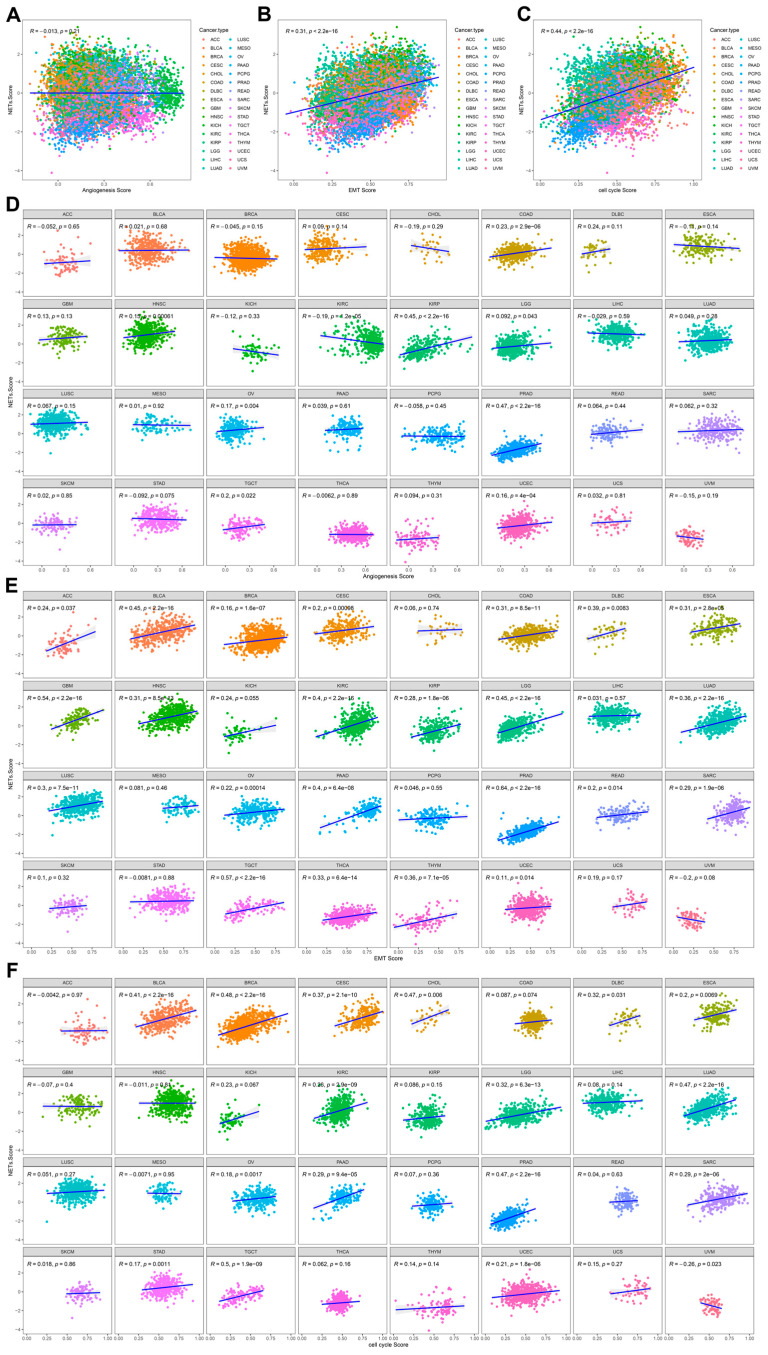
Correlation between the inflammation-related gene signature and key cancer hallmarks across multiple cancer types. Scatter plots showing pan-cancer correlations of inflammation scores with angiogenesis (**A**), EMT (**B**), and cell cycle activity (**C**), with R and *p*-values indicating statistical significance. Cancer-type-specific correlations of ferroptosis scores with angiogenesis (**D**), EMT (**E**), and cell cycle activity (**F**), demonstrating variability in inflammation-related influences on these processes across different tumors.

To investigate the biological significance of the inflammation-related gene signature, we analyzed its correlation with key cancer hallmarks, including angiogenesis, EMT, and cell cycle activity, across multiple tumor types. Fig. 6A shows a negative correlation between the risk score and angiogenesis (R = −0.13, *p* = 0.21, suggesting that inflammation may have a complex and cancer-type-dependent effect on tumor vascularization. In contrast, the risk score demonstrated a strong positive correlation with EMT (R = 0.31, *p* < 2.2 × 10^−^^16^) (Fig. 6B) and cell cycle activity (R = 0.44, *p* < 2.2 × 10^−^^16^) (Fig. 6C), indicating that tumors with higher inflammation-related gene expression tend to exhibit more aggressive characteristics, including increased metastatic potential and proliferative capacity. Fig. 6D further explores the correlation between the risk score and angiogenesis across different tumor types. While some cancers, such as HNSC and KIRC, displayed a significant positive correlation, others, such as LUAD and breast invasive carcinoma (BRCA), showed weaker associations. This suggests that inflammation-driven angiogenesis may be highly context-dependent. Fig. 6E examines the relationship between the risk score and EMT, revealing a strong positive correlation in cancers such as HNSC, KIRC, and PAAD, reinforcing the role of inflammation in tumor invasion and metastasis. Similarly, Fig. 6F shows the association between the risk score and cell cycle activity, with cancers such as HNSC, KIRC, and LIHC exhibiting a robust positive correlation, suggesting that inflammatory pathways contribute to uncontrolled tumor growth. These results indicate that the inflammation-related gene signature is closely linked to tumor progression, particularly by enhancing EMT and cell proliferation, which are key drivers of cancer aggressiveness. The variable effects on angiogenesis further emphasize that inflammation-mediated vascularization may differ by tumor type. Collectively, these findings suggest that targeting inflammation could be a potential strategy for tumor progression and metastasis in cancer therapy.

### 3.6 Functional Enrichment Analysis Reveals Distinct Biological Pathways Associated with the Inflammation-Related Risk Score

**Figure 7:**
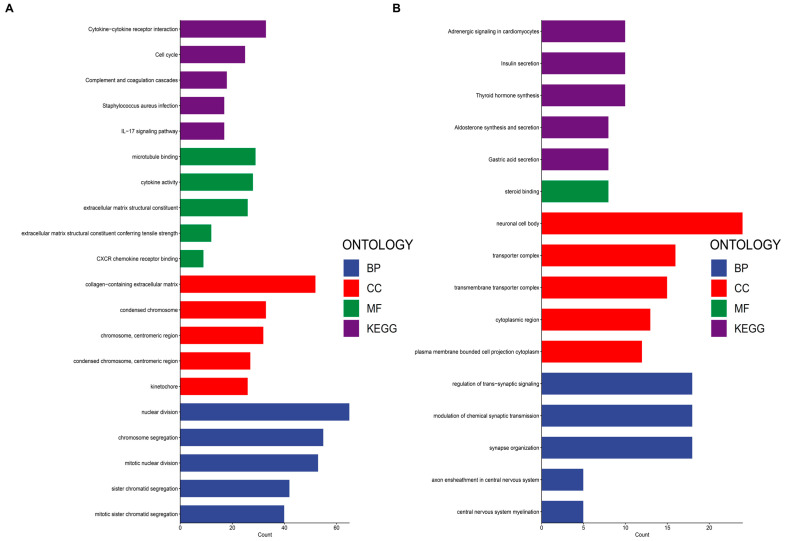
Functional enrichment analysis of differentially expressed genes between high- and low-risk groups based on the inflammation-related gene signature. (**A**) Enriched Gene Ontology (GO) terms and KEGG pathways in high-risk patients based on biological processes (BP), cellular components (CC), molecular functions (MF), and KEGG pathways. (**B**) Enriched terms and pathways in low-risk patients highlighting processes specific to hormonal signaling, metabolism, and development.

To explore the biological functions associated with the inflammation-related gene signature, we performed Gene Ontology (GO) and Kyoto Encyclopedia of Genes and Genomes (KEGG) enrichment analysis on differentially expressed genes (DEGs) between high- and low-risk groups. As shown in Fig. 7A, genes upregulated in the high-risk group were predominantly enriched in immune-related and tumor-promoting pathways. Notable pathways included cytokine–cytokine receptor interaction, IL-17 signaling, complement and coagulation cascades, and Staphylococcus aureus infection, suggesting a strong association between the high-risk score and chronic inflammation. Additionally, enrichment in cell cycle regulation, extracellular matrix structural organization, and CXCR chemokine receptor binding indicates that inflammation may contribute to tumor progression by promoting cell proliferation, immune evasion, and tissue remodeling. In contrast, Fig. 7B illustrates the functional enrichment of genes in the low-risk group, which were primarily associated with neurological and metabolic processes. Pathways such as regulation of trans-synaptic signaling, modulation of chemical synaptic transmission, and axon ensheathment suggest a potential neuroprotective role in tumors with lower inflammation-related gene expression. Moreover, metabolic pathways, including insulin secretion, aldosterone synthesis, and thyroid hormone synthesis, were significantly enriched, indicating that low-risk tumors may be more dependent on endocrine and metabolic regulation rather than inflammation-driven mechanisms. These findings highlight the dual role of inflammation in tumor biology, where high inflammation-related gene expression is linked to immune activation, extracellular matrix remodeling, and enhanced proliferative potential, while low inflammation levels are associated with neuronal and metabolic homeostasis. This suggests that targeting inflammation may be a viable therapeutic approach to mitigate tumor progression and improve clinical outcomes.

### 3.7 EXT1 Identified as the Key Gene Linking Inflammation and Glycosylation in Cancer

**Figure 8:**
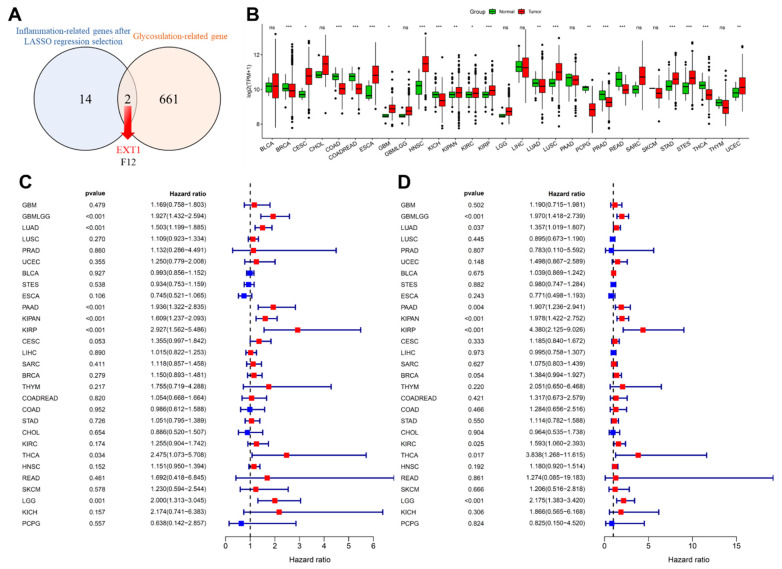
Identification and prognostic evaluation of inflammation-related and glycosylation-related genes. (**A**) Venn diagram showing the overlap between inflammation-related genes selected by LASSO regression and glycosylation-related genes, identifying EXT1 and F12 as common candidates. (**B**) Pan-cancer expression analysis of EXT1 using TCGA data, comparing tumor (red) and normal (green) tissues. (**C**) Forest plot illustrates the association between EXT1 expression and OS across multiple cancer types. (**D**) Forest plot depicting the correlation between EXT1 expression and DSS in different cancers. Statistical significance is indicated as ns: not significant, *: *p*-value < 0.05; **: *p*-value < 0.01; ***: *p*-value < 0.001.

To explore molecular connections between inflammation and glycosylation, we overlapped curated gene sets for these processes, both of which critically drive tumor progression, chronic inflammation reshapes the tumor immune microenvironment, while aberrant glycosylation modulates cell–cell interactions, immune evasion, and angiogenesis. Indeed, recent literature demonstrates that inflammatory signaling can induce N-glycosylation changes in immune and tumor cells [61], and that altered glycosylation contributes to tumor-induced immune suppression and metastasis [62]. As shown in Fig. 8A, EXT1 and F12 were the only two genes that overlapped between these two biological processes, with EXT1 emerging as the most critical gene linking inflammation and glycosylation. We next assessed the expression of EXT1 across cancers using TCGA datasets. EXT1 was significantly upregulated in multiple tumor types (Fig. 8B), including LUAD (*p* < 0.01), PAAD (ns), KIRP, (*p* < 0.001), and LGG (*ns*). In contrast, its expression was relatively lower in cancers such as breast invasive carcinoma (BRCA) and COAD, highlighting tumor-type-specific variation. To evaluate the prognostic significance of EXT1, we performed systematic survival analyses across TCGA cancers. As shown in Fig. 8C, high EXT1 expression was significantly associated with poor OS in LUAD (HR = 1.50, *p* < 0.001), PAAD (HR = 1.94, *p* < 0.001), KIRP (HR = 2.93, *p* < 0.001), and LGG (HR = 2.00, *p* = 0.001). Similarly, EXT1 expression predicted unfavorable DSS in LUAD (HR = 1.35, *p* = 0.037), PAAD (HR = 1.90, *p* < 0.004, KIRP (HR = 4.38, *p* < 0.001), and LGG (HR = 2.17, *p* < 0.001) (Fig. 8D). Although multiple cancers exhibited prognostic associations, LUAD and PAAD emerged as particularly compelling models for deeper investigation. Both are highly lethal epithelial malignancies with strong inflammatory and stromal components: LUAD is characterized by extensive immune infiltration, angiogenesis, and fibroblast activation, while PAAD develops within a profoundly desmoplastic and immune-excluded microenvironment. These features directly intersect with EXT1’s role in HS biosynthesis, which regulates growth factor availability (FGF, VEGF, TGF-β), stromal remodeling, and immune cell migration. Thus, LUAD and PAAD not only showed statistically significant EXT1 overexpression and poor prognosis, but also provided biologically relevant tumor contexts to investigate how EXT1 mediates glycosylation–inflammation crosstalk. Taken together, these findings identified LUAD and PAAD as the most relevant models, and they were therefore prioritized for subsequent validation and mechanistic analyses.

### 3.8 EXT1 Expression Correlates with Immune and Stromal Infiltration Across Different Cancers

**Figure 9:**
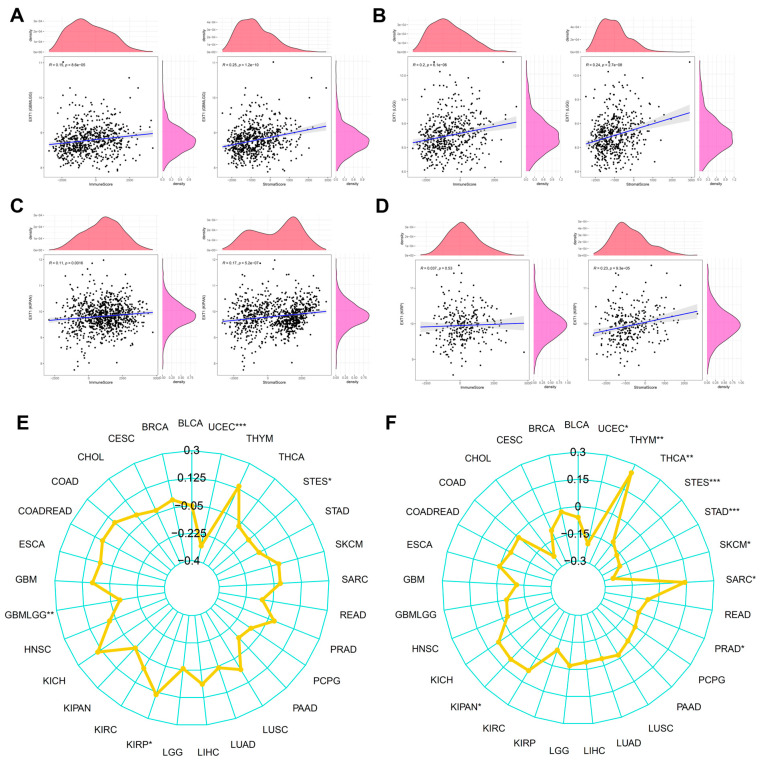
Correlation analysis of EXT1 expression with immune and stromal infiltration in specific cancer types. Correlation of EXT1 expression with immune and stromal scores in (**A**) glioblastoma multiforme/lower-grade glioma (GBMLGG), (**B**) lower-grade glioma (LGG), (**C**) kidney renal papillary cell carcinoma (KIRP), and (**D**) KIRC. (**E**) EXT1 expression is associated with microsatellite instability and tumor Mutation Burden Across Cancers. Correlation between EXT1 expression and MSI. (**F**) Correlation between EXT1 expression and TMB. Significant associations are marked (*p < 0.05, **p < 0.01, ***p < 0.001).

To explore the association between EXT1 expression and the TME, we conducted a comprehensive analysis across multiple cancer types. As shown in Fig. 9A–D, EXT1 expression exhibited a significant positive correlation with both immune and stromal infiltration scores, particularly in glioblastoma multiforme/lower-grade glioma (GBMLGG), lower-grade glioma (LGG), and kidney cancers. In GBMLGG (Fig. 9A), EXT1 showed a robust positive correlation with Immune Score (R = 0.15, *p* = 8.6e−05) and an even stronger correlation with Stromal Score (R = 0.25, *p* = 1.2e−10), suggesting that higher EXT1 expression is associated with an active immune and stromal microenvironment in gliomas. A similar trend was observed in LGG (Fig. 9B), where EXT1 correlated significantly with both Immune Score (R = 0.20, *p* = 6.1e−06) and Stromal Score (R = 0.24, *p* = 2.7e−08), reinforcing its relevance in modulating glioma TME composition. In KIRP (Fig. 9C), EXT1 was positively associated with Stromal Score (R = 0.17, *p* = 5.2e−07) and modestly with Immune Score (R = 0.11, *p* = 0.0016), suggesting a more prominent role in stromal remodeling, potentially through activation of cancer-associated fibroblasts and modulation of ECM dynamics. In contrast, in KIRC (Fig. 9D), EXT1 showed no significant correlation with Immune Score (R = 0.037, *p* = 0.53) but retained a moderate positive association with Stromal Score (R = 0.23, *p* = 9.3e−05), indicating that its function in KIRC may be more stromal-focused and less immune-involved. To further probe the relevance of EXT1 in tumor immunogenicity, we analyzed its correlation with MSI and TMB across cancer types. As illustrated in Fig. 9E,F, EXT1 expression was significantly associated with elevated MSI and TMB scores in cancers such as uterine corpus endometrial carcinoma (UCEC), thymoma (THYM), and gastrointestinal malignancies like stomach and esophageal cancers (STES, STAD). These associations are particularly noteworthy, as high MSI and TMB are well-established predictive markers for immunotherapy responsiveness. The positive correlation of EXT1 with these genomic instability indicators suggests that EXT1 may be involved in shaping a mutationally active and immunologically visible tumor landscape, possibly influencing DNA repair pathways or interacting with signaling cascades involved in replication stress and chromosomal instability. Mechanistically, EXT1 encodes an enzyme essential for HS biosynthesis, and altered EXT1 activity may impact cytokine and chemokine presentation, ECM organization, and receptor-ligand signaling at the cell surface. These changes could directly influence immune cell recruitment, stromal activation, and inflammatory pathway engagement within the TME. The strong stromal and immune associations observed in gliomas and renal cancers imply that EXT1 may be involved in tumor–stroma crosstalk, potentially contributing to immune evasion, ECM remodeling, and metastasis. Collectively, these findings underscore the context-dependent immunomodulatory and stromal regulatory roles of EXT1 across diverse cancer types. Its strong associations with immune/stromal infiltration, MSI, and TMB highlight EXT1 as a potential biomarker of immune activity and genomic instability, as well as a promising therapeutic target in inflammation-driven and immune-evasive malignancies.

### 3.9 EXT1 Expression Is Associated with Immune Cell Infiltration and Inflammatory Signaling Pathways Across Multiple Cancers

**Figure 10:**
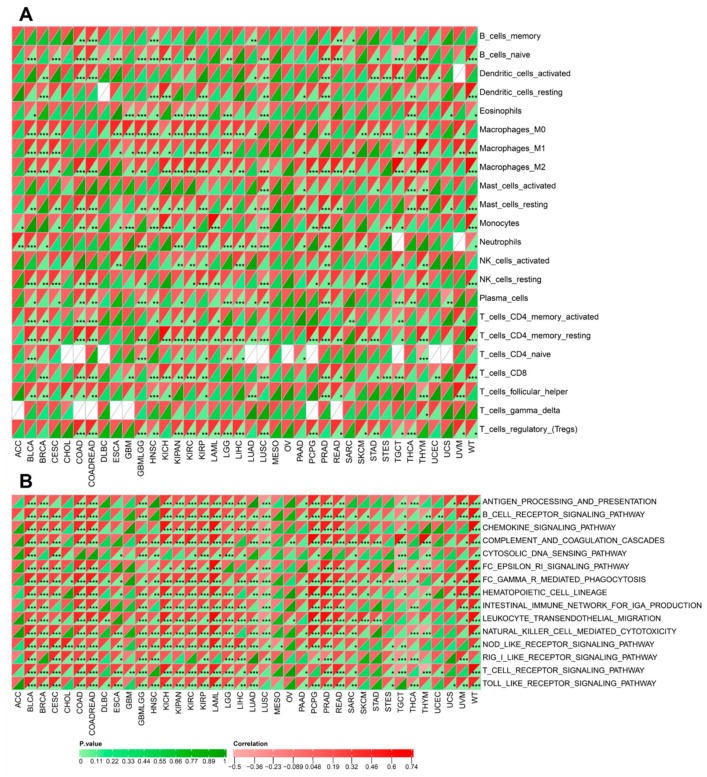
Correlation of EXT1 expression with immune cell infiltration and immune-inflammatory pathway activity across cancers. (**A**) Spearman correlation analysis between EXT1 expression and infiltration levels of distinct immune cell subsets, including B cells, T cells (CD4^+^, CD8^+^, follicular helper, regulatory), macrophages (M0, M1, M2), dendritic cells, NK cells, mast cells, neutrophils, eosinophils, monocytes, and plasma cells across pan-cancer cohorts. (**B**) Spearman correlation between EXT1 expression and immune-related signaling pathways, including antigen processing and presentation, B cell receptor signaling, chemokine signaling, complement cascade, cytosolic DNA sensing, Fc epsilon receptor signaling, hematopoietic cell lineage, leukocyte transendothelial migration, NK cell-mediated cytotoxicity, NOD-like receptor signaling, RIG-I-like receptor signaling, T cell receptor signaling, and Toll-like receptor pathways. Correlation strength is indicated by the heatmap scale (green = negative correlation, red = positive correlation). Statistical significance was determined by Spearman’s test: *p < 0.05, **p < 0.01, ***p < 0.001.

To further elucidate the immunological relevance of EXT1 in cancer, we examined its correlation with immune cell infiltration and the activation of inflammatory signaling pathways across diverse tumor types. As shown in Fig. 10A, EXT1 expression exhibited cancer-type-specific associations with various immune cell populations. In kidney renal papillary cell carcinoma (KIRP) and lower-grade glioma (LGG), EXT1 expression was strongly and positively correlated with M2-polarized macrophages, activated dendritic cells, and regulatory T cells (Tregs), all of which are key mediators of immune suppression in the TME. These findings suggest that EXT1 may contribute to establishing or sustaining an immunosuppressive niche that promotes tumor progression in specific cancer contexts. Conversely, in malignancies such as LUAD and breast invasive carcinoma (BRCA), the correlation between EXT1 and immune infiltration was comparatively weak, indicating a more variable or context-dependent role for EXT1 in modulating tumor immunity. Further pathway-level analysis revealed significant positive associations between EXT1 expression and major immune-inflammatory signaling cascades (Fig. 10B). Notably, EXT1 was correlated with pathways such as B cell receptor signaling, T cell receptor signaling, and Toll-like receptor pathways, implicating its involvement in both innate and adaptive immune regulation. In inflammation-associated tumors like COAD and LIHC, EXT1 expression showed strong correlations with genes involved in leukocyte transendothelial migration, chemokine signaling, and complement activation, suggesting a role in orchestrating inflammatory cell recruitment and activation. Collectively, these findings highlight EXT1 as a potential modulator of the immune landscape within tumors. Its consistent association with both immunosuppressive cell infiltration such as Tregs, M2 macrophages, and pro-inflammatory signaling suggests a complex dual role in immune evasion and inflammation-driven oncogenesis. These properties underscore EXT1’s potential as a biomarker of tumor immune status and a promising candidate for immunomodulatory therapeutic strategies in inflammation-driven cancers.

### 3.10 Single-Cell Transcriptomic Profiling of EXT1 in PAAD, Liver Metastasis, and Lung Tissue

**Figure 11:**
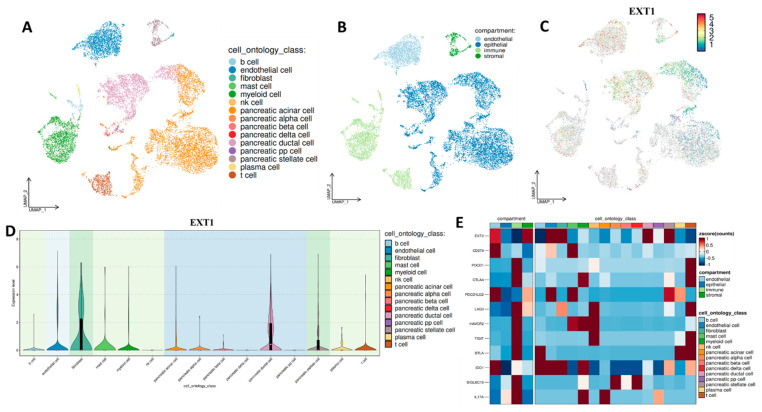
Single-cell transcriptomic analysis of EXT1 and its association with immune checkpoint and exhaustion markers in PAAD. (**A**) UMAP clustering of pancreatic adenocarcinoma (PAAD) single-cell transcriptomes, annotated by cell ontology classes including ductal, acinar, stellate, immune, and stromal cell types. (**B**) Cells are grouped into four major compartments: epithelial, endothelial, immune, and stromal. (**C**) UMAP feature plot showing EXT1 expression, with enrichment observed in ductal, stellate, and fibroblast compartments. (**D**) Violin plots showing EXT1 expression across annotated cell types, confirming the highest expression in stromal and ductal cells. (**E**) Heatmap of EXT1 expression and immune checkpoint/exhaustion markers, including CD274 (PD-L1), PDCD1 (PD-1), CTLA4, LAG3, HAVCR2 (TIM-3), TIGIT, BTLA, IDO1, and SIGLEC15. EXT1 expression shows positive associations with multiple inhibitory pathways, suggesting that EXT1 contributes to an immune-suppressive and immune-cold microenvironment in PAAD.

**Figure 12:**
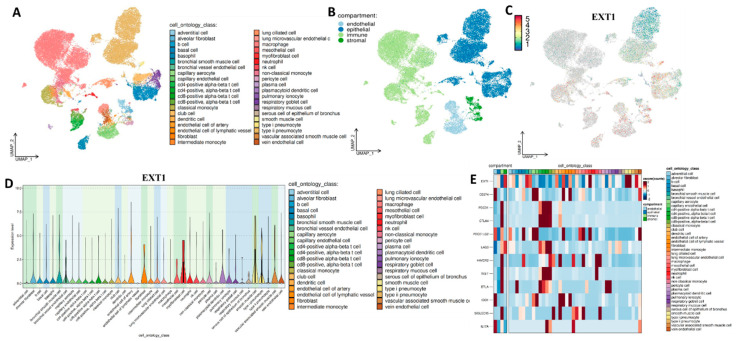
Single-cell transcriptomic analysis of EXT1 and its association with immune checkpoint and exhaustion markers in LUAD. (**A**) UMAP clustering of LUAD single-cell transcriptomes, annotated by detailed cell ontology classes. (**B**) Cells are stratified into four major compartments: epithelial, endothelial, immune, and stromal. (**C**) UMAP feature plot showing EXT1 expression, with enrichment observed in epithelial, endothelial, and stromal populations. (**D**) Violin plots displaying EXT1 expression across cell types, confirming compartment-specific localization. (**E**) Heatmap of EXT1 expression in relation to immune checkpoint and exhaustion markers, including CD274 (PD-L1), PDCD1 (PD-1), CTLA4, LAG3, HAVCR2 (TIM-3), TIGIT, BTLA, IDO1, and SIGLEC15. EXT1 expression positively associates with multiple inhibitory pathways, suggesting that EXT1 contributes to the establishment of an immune-cold TME in LUAD.

To elucidate the cell-type-specific expression landscape of EXT1, we performed single-cell RNA-seq analysis using publicly available datasets for PAAD and LUAD. Our analysis in PAAD, EXT1 expression was localized predominantly to ductal epithelial cells and pancreatic stellate cells, the key drivers of the desmoplastic and fibrotic stroma that defines pancreatic cancer (Fig. 11A–D). Pancreatic stellate cells, once activated, secrete extracellular matrix components and remodel the TME into a dense, hypoxic, and immune-excluded niche [63,64]. The presence of EXT1 in these stromal compartments suggests that HS proteoglycan remodeling contributes directly to fibrosis, barrier formation, and sequestration of immune effector cells. Consistent with this, EXT1-high PAAD cells were enriched for TGF-β and ECM-related pathways, both central to stromal activation and immune exclusion. Moreover, single-cell correlation analysis (Fig. 11E) demonstrated that EXT1 was co-expressed with checkpoint and exhaustion markers, including PD-L1, CTLA4, TIM-3, and SIGLEC15, the latter of which has recently been identified as a non-redundant suppressive molecule in PD-1/PD-L1-resistant tumors. This suggests that EXT1 not only contributes to structural remodeling of the PAAD stroma but also aligns with transcriptional programs that enforce immune evasion. Biologically, this is consistent with recent reports showing that HS modifications regulate TGF-β gradients [65], fibroblast activation, and stromal immune cross-talk in PAAD, reinforcing EXT1’s dual role as both a glycosylation enzyme and an indirect regulator of inflammation-driven immune suppression. Further, our analysis of LUAD revealed that EXT1 expression was concentrated in alveolar epithelial cells and fibroblasts, two compartments central to tumor initiation and progression (Fig. 12A–D). In LUAD, alveolar epithelial cells are the origin of most adenocarcinomas, and their interaction with the stromal compartment dictates angiogenesis, immune infiltration, and therapy resistance. The enrichment of EXT1 in these populations is biologically consistent with its function as a heparan-sulfate (HS) glycosyltransferase, which polymerizes the HS backbone and regulates the bioavailability of cytokines and growth factors such as FGF, VEGF, and TGF-β [66,67]. These growth factors not only drive angiogenesis but also remodel the immune landscape by recruiting suppressive myeloid populations and attenuating effector T-cell trafficking. HS proteoglycans produced by EXT1-high fibroblasts further create a matrix scaffold that supports vascular sprouting and modulates chemokine gradients, thereby shaping the spatial distribution of immune cells. This biological framework aligns with our single-cell correlation findings (Fig. 12E), where EXT1 expression is positively associated with immune checkpoint and exhaustion markers, including PD-L1 (CD274), PD-1 (PDCD1), CTLA4, LAG3, HAVCR2 (TIM-3), and TIGIT. Given that N-glycosylation stabilizes PD-L1 and PD-1 proteins, enhancing their surface expression and immunosuppressive capacity, the observed correlation suggests that EXT1 indirectly potentiates checkpoint pathways by sustaining a glycosylation-rich microenvironment. These findings are further reinforced by published evidence that galectins, canonical glycan-binding proteins, orchestrate immunosuppressive programs through T-cell apoptosis, Treg expansion, and pro-angiogenic cytokine regulation [68,69]. Together, these mechanisms provide a strong biological rationale that EXT1 expression in LUAD contributes to an immune-cold phenotype, characterized by stromal activation, checkpoint engagement, and reduced cytotoxic T-cell activity. Together, the PAAD (Fig. 11) and LUAD (Fig. 12) single-cell datasets provide high-resolution validation of our multi-omics results. By mapping EXT1 expression to epithelial and stromal compartments and linking it to checkpoint/exhaustion programs, these analyses demonstrate that EXT1 integrates glycosylation with inflammation-driven immune suppression. These findings underscore EXT1’s role as a compartment-specific mediator of tumor aggressiveness and immune evasion, supporting its potential as a biomarker and therapeutic candidate at the glycosylation–inflammation interface.

### 3.11 Tumor-Type-Specific Methylation Landscapes of EXT1 Highlight Distinct Epigenetic Regulation Patterns in PAAD and LUAD

**Figure 13:**
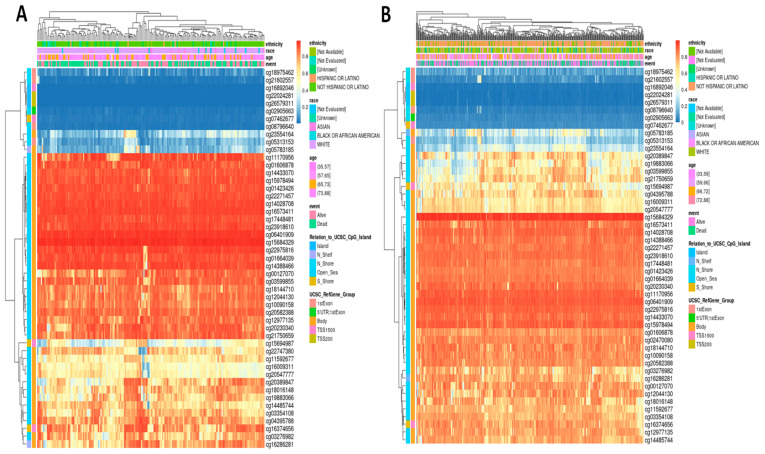
**EXT1-associated DNA methylation landscape across PAAD and LUAD.** (**A**) Heatmap showing the top EXT1-correlated CpG sites in PAAD, displayed with unsupervised hierarchical clustering of both CpG sites (rows) and patient samples (columns). β-values are represented on a blue-to-red scale (blue = hypomethylation; red = hypermethylation). Clinical annotations above the heatmap include ethnicity, race, age group, and survival status (alive or dead). Additional annotation tracks denote CpG island context (Island, Shore, Shelf, Open Sea) and UCSC RefGene groups (5′UTR, 1st Exon, TSS200, TSS1500, Body). (**B**) Heatmap of the top EXT1-correlated CpG sites in LUAD, generated using the same clustering and annotation scheme as in Panel A. The methylation distribution highlights interpatient heterogeneity and the genomic positions of CpG sites relative to CpG islands and gene regulatory regions.

To further elucidate the regulatory landscape of EXT1, we examined DNA methylation profiles of CpG sites correlated with EXT1 expression across PAAD and LUAD. For each tumor type, the top variably methylated CpG loci associated with EXT1 (Pearson r threshold ≥ 0.3, *p* < 0.01) were extracted and visualized via unsupervised hierarchical clustering heatmaps (Fig. 10). In PAAD (Fig. 13A), the majority of EXT1-linked CpG sites exhibited widespread hypomethylation, particularly within promoter-adjacent regions (CpG islands and TSS1500). Notably, patients classified into the hypomethylated subgroup were more frequently associated with poor prognosis (deceased status), implicating epigenetic derepression of EXT1 as a potential driver of pancreatic tumor aggressiveness. Conversely, LUAD (Fig. 13B) demonstrated high interpatient variability in EXT1 methylation status. No dominant methylation pattern was observed, though subsets of hypomethylated CpG sites in 1stExon and TSS200 regions were enriched in younger and non-White patient groups, pointing to a potential demographic-linked epigenetic divergence. The relatively diffuse clustering suggests that EXT1 methylation in LUAD may be governed by heterogeneous tumor-intrinsic and microenvironmental influences. These results highlight the tumor-type-specific epigenetic regulation of EXT1, with distinct methylation architectures that may underlie its differential expression and functional deployment in PAAD and LUAD. These findings reinforce the importance of integrating multi-layered genomic context when evaluating EXT1 as a pan-cancer biomarker or therapeutic target.

### 3.12 MetaCore Pathway Enrichment Reveals Functional Roles of EXT1 Co-Expression in PAAD and LUAD

**Figure 14:**
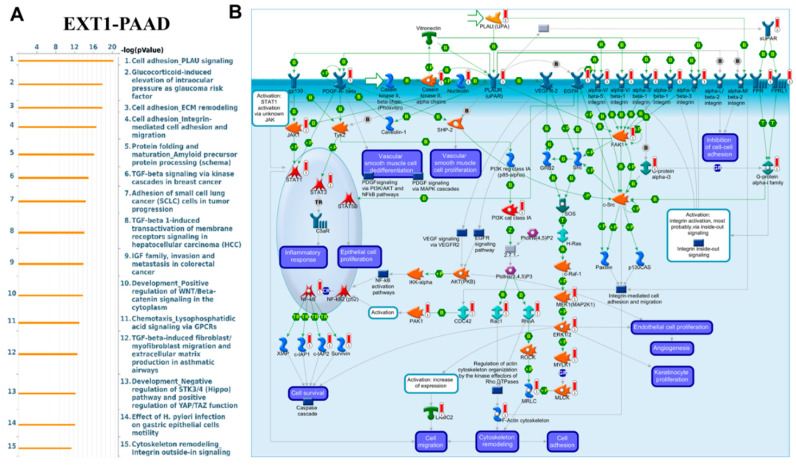
**MetaCore pathway enrichment analysis of EXT1-correlated genes in PAAD.** Top-ranked pathways enriched in EXT1-associated gene networks include cell adhesion, ECM remodeling, integrin-mediated adhesion and migration, TGF-β signaling, and lysophosphatidic acid (LPA) receptor signaling. These cascades regulate fibroblast activation, extracellular matrix deposition, angiogenesis, and cytoskeletal remodeling, processes central to pancreatic tumor progression and stromal remodeling. (**A**) Ranked pathways by −log(*p*-value), and (**B**) representative signaling modules identified by MetaCore, highlighting interactions between growth factors, integrins, STAT/PI3K/AKT, and Rho GTPase effectors.

**Figure 15:**
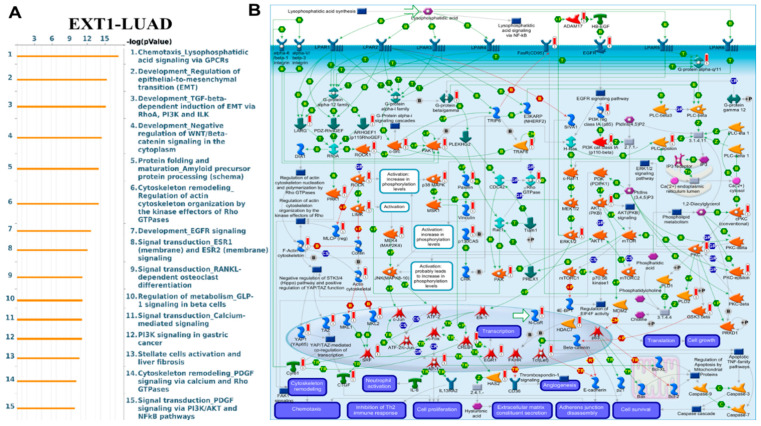
MetaCore pathway enrichment analysis of EXT1-correlated genes in LUAD. Enriched signaling pathways associated with EXT1 expression include lysophosphatidic acid (LPA) signaling via GPCRs, EMT, TGF-β–dependent EMT regulation, EGFR/ERBB signaling, and PI3K–AKT cascades. These networks converge on cytoskeletal remodeling, cell adhesion, angiogenesis, and immune modulation, highlighting the role of EXT1 in promoting epithelial remodeling and immune evasion in LUAD. (**A**) Ranked enrichment of pathways by −log(*p*-value), while (**B**) representative MetaCore pathway maps illustrating EXT1-linked regulation of growth factor signaling, Rho GTPase effectors, and integrin-associated cytoskeletal dynamics.

To investigate the potential biological roles of EXT1 in cancer, we performed MetaCore pathway enrichment analysis using co-expressed gene signatures from both PAAD and LUAD. MetaCore analysis revealed a highly coherent enrichment of pathways that regulate cell adhesion, ECM remodeling, integrin-mediated signaling, lysophosphatidic acid (LPA) chemotaxis, TGF-β signaling, EGFR–PI3K–AKT–MAPK cascades, cytoskeletal remodeling via Rho GTPases, and EMT (Fig. 14 and Fig. 15) [70,71,72]. These enrichments are mechanistically consistent with the biochemical role of EXT1, which functions as a glycosyltransferase that, together with EXT2, polymerizes HS chains on core proteoglycans. HS proteoglycans such as syndecans and glypicans act as broad co-receptors that bind growth factors, morphogens, and cytokines, thereby increasing their stability, controlling diffusion, and facilitating presentation to high-affinity signaling receptors. Through this scaffolding function, HS determines the strength, duration, and spatial range of signaling events that drive both stromal remodeling and immune regulation. 

One of the top enriched pathways was LPA receptor signaling via GPCRs. As shown in Fig. 14, this pathway controls chemotaxis, actin cytoskeleton remodeling, focal adhesion turnover, EMT, and survival through Gα12/13–Rho/ROCK, Gαq–PLC–Ca^2^^+^, and Gαi–PI3K/AKT/ERK signaling branches [73,74]. These cascades have well-established roles in tumor invasion, inflammatory remodeling of the TME and resistance to therapy in both LUAD and PAAD. In pancreatic cancer specifically, the autotaxin–LPA axis has been shown to modulate immune infiltration and fibrosis; inhibiting this axis improves immune responses [75,76]. Because HS organizes ligand–receptor complexes, EXT1-dependent HS biosynthesis likely enhances LPA receptor clustering and stabilizes downstream signals. TGF-β signaling was another major enrichment, particularly relevant to fibrosis and immune exclusion. As visualized in Fig. 14, HS binds TGF-β family members and regulates their gradient formation, thereby controlling fibroblast activation, desmoplasia, and stromal remodeling [77,78,79]. In PAAD, where EXT1 was concentrated in ductal epithelial cells and stellate fibroblasts (Fig. 11), this pathway explains the observed desmoplastic and immune-excluded niche. EGFR/ERBB–PI3K/AKT/MAPK and integrin–FAK–Rho GTPase axes were also prominently enriched (Fig. 15). HS proteoglycans present ligands such as EGF and FGF to their receptors, enhancing dimerization and clustering [80,81]. This potentiates proliferative and survival signaling, while integrin–FAK pathways reinforce cytoskeletal remodeling and EMT. These pathways converge on actin reorganization, migration, and angiogenesis, which are hallmarks of LUAD and PAAD stromal biology. MetaCore also highlighted links between EXT1 and immune regulation. Glycosylation directly modulates immune checkpoints; N-glycosylation stabilizes PD-L1 and PD-1 proteins at the plasma membrane, increasing their immunosuppressive activity [82,83]. This provides a molecular explanation for the strong correlation of EXT1 with checkpoint and exhaustion markers (PD-L1, PD-1, CTLA4, LAG3, TIM-3, TIGIT) observed in our single-cell analysis. Taken together, these results indicate that EXT1 establishes a heparan-sulfate–dependent signaling hub that amplifies LPA, TGF-β, EGFR, and integrin pathways to drive stromal activation, angiogenesis, EMT, and cytoskeletal remodeling, while simultaneously stabilizing immune checkpoints and enabling galectin- and SIGLEC-mediated immune suppression. The MetaCore pathway maps (Fig. 14 and Fig. 15) thus provide a mechanistic bridge connecting glycosylation to inflammation, supporting our single-cell findings in PAAD and LUAD that EXT1-high epithelial and stromal compartments orchestrate stromal remodeling and immune evasion. The complete MetaCore pathway enrichment results, including full enrichment tables and expanded pathway diagrams for both PAAD and LUAD, are provided in the Supplementary Figures (Supplement Figs. S1–S4 and Supplement Tables S1 and S2).

### 3.13 Docking and Dynamics Simulation Reveal 17-AAG as a Potential EXT1 Modulator

**Figure 16:**
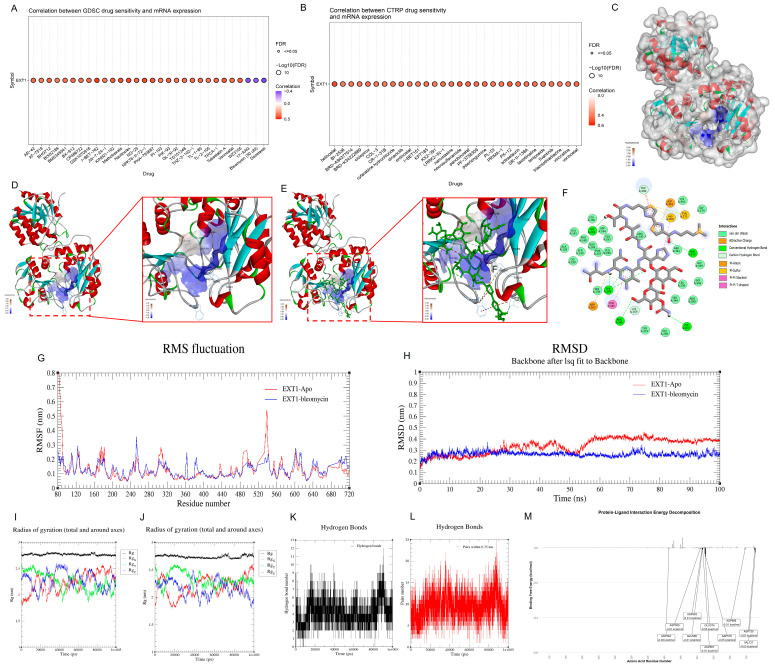
**Molecular docking and molecular dynamics analyses of the EXT1–17-AAG complex**. (**A**) Correlation between EXT1 expression and compound sensitivity in the GDSC database. (**B**) Correlation between EXT1 expression and compound sensitivity in the CTRP database. (**C**) Surface representation of the EXT1 protein structure used for docking. (**D**) Predicted binding pocket of EXT1 showing the ligand-accessible cavity. (**E**) Docked pose of 17-AAG within the EXT1 binding pocket (3D view). (**F**) Root-mean-square deviation (RMSD) of the protein backbone over a 100-ns simulation comparing apo EXT1 and the EXT1–17-AAG complex. (**G**) Root-mean-square fluctuation (RMSF) of residues in apo EXT1 versus the EXT1–17-AAG complex. (**H**) Two-dimensional interaction diagram showing the specific amino acid residues interacting with 17-AAG. (**I**) Radius of gyration (Rg) of apo EXT1 across the simulation. (**J**) Radius of gyration (Rg) of the EXT1–17-AAG complex across the simulation. (**K**) Number of hydrogen bonds between EXT1 and 17-AAG throughout the simulation using a double-cutoff criterion. (**L**) Number of hydrogen bonds between EXT1 and 17-AAG using a single-cutoff criterion. (**M**) Per-residue free energy decomposition from the MM–PBSA calculation, highlighting the top residues contributing to ligand binding.

To further elucidate the therapeutic potential of EXT1 inhibition, we employed a combined approach of pharmacogenomic screening, molecular docking, and molecular dynamics simulations. GSCA-based drug correlation analysis identified 17-AAG, along with Bleomycin and Docetaxel, as negatively correlated compounds with EXT1 expression (Fig. 16A,B), suggesting a potential vulnerability in EXT1-high expressing tumors. Among these, 17-AAG demonstrated the most favorable binding characteristics, warranting in-depth structural and dynamic evaluation. We first visualized the spatial and electrostatic structure of EXT1 (Fig. 16C), which revealed a distinct hydrophobic groove, a potential drug-binding cavity. Through molecular docking, 17-AAG was predicted to fit stably within this pocket (Fig. 16D,E), engaging in a network of hydrogen bonds, van der Waals contacts, and Pi-Sigma interactions. Specifically, residues such as Tyr196, Gly343, Arg341, and Ser344 formed strong polar interactions with the ligand, while hydrophobic stabilization was achieved through residues like His300, Leu236, and Arg340. These interactions are clearly illustrated in the 2D interaction map (Fig. 16H), highlighting the diverse non-covalent forces maintaining ligand stability. To assess whether these interactions are dynamically preserved, we further performed 100 ns molecular dynamics (MD) simulations. The RMSD profile (Fig. 16F) of the EXT1–17-AAG complex remained within a stable range (0.25–0.30 nm) after initial equilibration, indicating that the complex maintains its structural integrity over time. In addition, the radius of gyration (Rg) (Fig. 16I,J) showed minimal fluctuation, confirming the compactness and global stability of the protein-ligand complex. We further analyzed the residue-specific flexibility through RMSF profiling (Fig. 16G,H). Notably, residues within the 260–360 region, which comprise the predicted binding site, displayed increased fluctuation upon ligand binding when compared to the unbound (apo) form. This suggests a ligand-induced conformational adjustment, potentially modulating the functional state of EXT1. Such localized flexibility may reflect a mechanism through which 17-AAG disrupts EXT1 activity, a hypothesis supported by similar observations in other Hsp90-related interactions. In addition to structural stability, we examined non-covalent bonding patterns over the simulation trajectory. The number of hydrogen bonds between EXT1 and 17-AAG was consistently maintained throughout the simulation, ranging between 4 to 6 (Fig. 16K,L), reinforcing the stability of the interaction network. Importantly, key residues such as Tyr196 and Arg341 contributed persistently to hydrogen bonding, underscoring their potential as pharmacophores for future ligand optimization. To pinpoint the energetic contributors to binding, we conducted a per-residue energy decomposition analysis using MM-PBSA (Fig. 16M). Here, residues like Tyr196, Arg341, and Phe345 exhibited the highest negative free energy contributions, aligning with our interaction and MD analyses. This suggests these residues are central to ligand anchoring and could be prioritized in structure-based drug design efforts targeting EXT1. Together, these results provide compelling evidence that 17-AAG binds stably and specifically to EXT1, induces localized conformational changes, and is supported by strong non-covalent interactions and favorable energetics. These findings not only highlight 17-AAG as a potential repurposable inhibitor of EXT1 but also offer a structural framework for developing novel analogs or combination strategies in EXT1-associated malignancies.

### 3.14 Clinical Validation of EXT1 Upregulation in PAAD and LUAD by Immunohistochemistry

**Figure 17:**
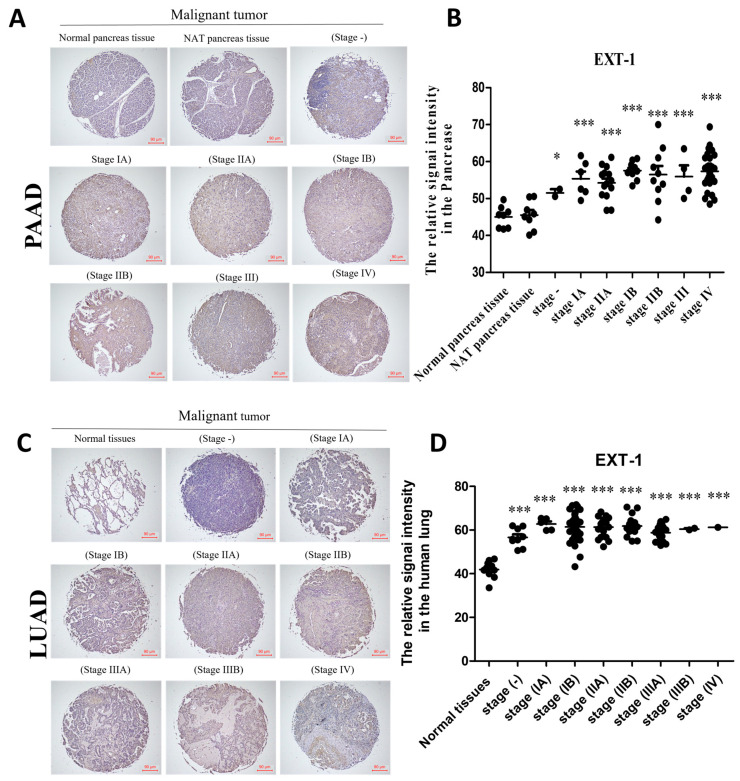
IHC staining of EXT1 in tumor tissues from PAAD and LUAD. (**A**) Representative IHC images of EXT1 expression in normal pancreatic tissues. (**B**) Representative IHC images of EXT1 expression in PAAD tissues at different clinical stages (Stage I–IV). EXT1 staining was mainly localized to the cytoplasm of ductal epithelial and stromal compartments, with gradually increased expression observed from early to late-stage tumors. (**C**) IHC images of EXT1 expression in normal lung tissues. (**D**) EXT1 IHC staining in LUAD samples across pathological stages (IA, IB, IIA, IIB, IIIA, IIIB, and IV). EXT1 expression was primarily detected in alveolar epithelial and fibroblast cells, with markedly elevated staining intensity in advanced-stage LUAD tissues. Statistical analysis confirmed significant upregulation of EXT1 in malignant tissues compared to normal controls (***p < 0.001). Data are presented as dot plots with mean ± SD, and statistical significance was determined using an unpaired two-tailed *t*-test (*p = 0.0261). Scale bar = 50 μm. Representative images are shown for each group.

To validate our transcriptomic findings at the protein level, we performed IHC staining using clinically annotated tissue microarrays for PAAD (PA1001e, 49 cases/98 cores) and LUAD (LC1203A, 120 cases/120 cores). These arrays provided a wide representation of tumor stages and histological subtypes, as well as matched normal pancreatic and lung tissue controls that served as critical baselines for comparison. In PAAD tissues (Fig. 17A), EXT1 protein was predominantly localized to the cytoplasm of ductal epithelial tumor cells and pancreatic stellate cells (PSCs), the major drivers of desmoplasia. EXT1 expression showed a progressive increase from early (Stage I–II) to advanced (Stage III–IV) disease, with strong staining in stromal regions of late-stage tumors. Biologically, this is significant because PSC activation is central to the development of the dense, fibrotic stroma in PAAD, which increases interstitial pressure, reduces vascular perfusion, and excludes cytotoxic T cells. EXT1-mediated HS biosynthesis likely regulates fibroblast growth factor (FGF) and TGF-β gradients, thereby fueling fibrosis and immunosuppressive cross-talk between PSCs, macrophages, and Tregs. This supports the idea that EXT1 is not just a marker but an active driver of the immune-cold, therapy-resistant PAAD microenvironment.

In LUAD tissues (Fig. 17B), EXT1 was largely absent in normal alveolar epithelial and fibroblast compartments, but strongly expressed in tumor cells and fibroblasts across pathological stages IA–IV. EXT1 levels were significantly higher in malignant tissues compared to adjacent normal lung controls (*p < 0.001; n = 110), and staining intensity increased with advancing stage. Functionally, LUAD originates from alveolar type II epithelial cells, which rely on growth factor signaling for transformation. By polymerizing HS chains, EXT1 facilitates VEGF- and FGF-dependent angiogenesis, promotes fibroblast activation, and remodels the extracellular matrix to create niches favorable for tumor invasion. Moreover, stromal EXT1 expression aligns with LUAD’s high fibroblast–immune cell interplay, where HS proteoglycans stabilize PD-L1 and other checkpoint molecules, supporting immune evasion. Mechanistically, these results confirm that EXT1 integrates glycosylation and inflammation within the TME. Its activity in stromal and epithelial compartments enables tumor cells to exploit HS-mediated signaling for survival and expansion. By shaping ECM stiffness, vascularization, and checkpoint stabilization, EXT1 contributes to fibrosis in PAAD and angiogenesis in LUAD, two distinct but equally immunosuppressive tumor ecosystems. Taken together, these findings demonstrate that EXT1 is consistently upregulated in late-stage PAAD and LUAD, validated across large and clinically diverse tissue microarrays. Its dual role in structural remodeling (ECM, fibrosis, angiogenesis) and immune regulation (checkpoint stabilization, immune exclusion) underscores EXT1 as both a biomarker of tumor aggressiveness and a promising therapeutic target at the glycosylation–inflammation interface.

## 4 Discussion

Cancer progression is orchestrated by the interplay between inflammation, stromal remodeling, and immune evasion. Glycosylation has emerged as a central modifier of these processes, yet the specific glycosyltransferases that directly bridge inflammation and tumor biology remain incompletely defined. Previous studies have established galectins as glycan-binding “readers” that connect glycosylation to immune suppression, T-cell apoptosis, regulatory T-cell expansion, and angiogenesis. However, galectins interpret glycan structures without directly synthesizing them [8,84]. In contrast, EXT1 functions as a glycan “writer,” encoding a glycosyltransferase essential for HS biosynthesis, which regulates growth factor gradients, ECM organization, and immune signaling [85,86]. Despite this central role, EXT1 has not been systematically investigated in the context of cancer-associated inflammation. By focusing on EXT1, our study addresses this critical gap, shifting attention from glycan readers to glycan writers. This mechanistic reframing underscores the novelty of our findings and provides a new framework for glycosylation biology in tumor progression. We first performed a pan-cancer transcriptomic screen, integrating curated inflammation- and glycosylation-related gene sets to identify potential molecular bridges between these processes. Using a LASSO Cox regression model, we constructed an inflammation-related prognostic signature capable of stratifying patients into high- and low-risk groups, with significant differences in OS, DSS, and PFI across TCGA cohorts (Fig. 2, Fig. 3 and Fig. 4). Importantly, EXT1 emerged as one of the top contributors to this signature, linking its expression directly to adverse clinical outcomes.

To further validate this finding, we performed an overlap analysis that revealed only two candidates, EXT1 and F12, at the intersection of inflammation and glycosylation (Fig. 2). Among these, EXT1 was the most consistently expressed and biologically plausible regulator. Extending this to pan-cancer profiling, we observed that EXT1 was broadly upregulated across multiple tumor types (Fig. 5), with high expression strongly predicting poor OS and DSS (Fig. 6 and Fig. 7). These results positioned EXT1 as a candidate of high clinical relevance. Building upon these observations, we next asked whether EXT1 exerted context-specific roles in distinct TME. Pan-cancer survival analyses indicated that its prognostic impact was strongest in LUAD and PAAD (Fig. 8C,D), two inflammation-driven cancers with fibroblast-rich stroma [87]. This justified focusing subsequent analyses on these tumor types. In PAAD, we found EXT1 expression enriched in ductal epithelial cells and PSCs by single-cell RNA-seq (Fig. 11A–D). Because activated PSCs remodel ECM, induce hypoxia, and block immune infiltration [88,89,90], EXT1 localization here suggests that HS biosynthesis promotes fibrosis and barrier formation. Consistently, EXT1-high PSCs co-expressed immune checkpoints including PD-L1, CTLA4, TIM-3, and SIGLEC15 (Fig. 11E), directly linking glycosylation to immunosuppressive signaling [91]. In LUAD, EXT1 was enriched in alveolar epithelial cells and cancer-associated fibroblasts (CAFs) (Fig. 12A–D). These compartments regulate angiogenesis and stromal–immune crosstalk. EXT1-dependent HS chains modulated VEGF and FGF signaling, recruited suppressive myeloid cells, and stabilized vascular sprouting [92,93]. Single-cell correlation analysis further showed co-expression with PD-L1, PD-1, CTLA4, LAG3, TIM-3, and TIGIT (Fig. 12E). Since N-glycosylation stabilizes PD-1/PD-L1 proteins [7], these results reinforce EXT1’s role in enhancing checkpoint activity.

To complement single-cell findings, we next analyzed immune infiltration in bulk datasets. EXT1 expression was positively correlated with immunosuppressive cells such as M2 macrophages, Tregs, and activated dendritic cells, but showed little association with effector CD8^+^ T cells or NK cells (Fig. 9A–D and Fig. 10A). This imbalance suggests that EXT1-high tumors adopt an immune-cold phenotype, a hallmark of immunotherapy resistance. Further enrichment analysis confirmed that EXT1 was linked to immune-inflammatory signaling, including TCR, BCR, and TLR pathways as well as leukocyte transendothelial migration (Fig. 10B). These pathways reinforce its role as a regulator of both innate and adaptive immunity, frequently exploited by tumors to evade immune surveillance. To further probe its regulation, we investigated genomic and epigenomic features. EXT1 expression was associated with MSI and TMB in several cancers. In UCEC and STES, EXT1 positively correlated with MSI/TMB (Fig. 9E,F), suggesting a potential link to immunogenic phenotypes. Conversely, in gliomas and renal cancers, EXT1 was negatively associated with these markers, implying alternative oncogenic roles such as metabolic or epigenetic reprogramming. At the epigenetic level, DNA methylation profiling revealed hypomethylation of EXT1 promoter CpG islands in PAAD and LUAD, correlating with higher expression and poor outcomes (Fig. 13A,B). Thus, epigenetic derepression provides an additional regulatory layer reinforcing EXT1 activation. To clarify the biological consequences of EXT1 activation, we next performed MetaCore enrichment of EXT1-correlated genes in LUAD and PAAD. The results showed strong convergence on LPA receptor signaling, TGF-β cascades, EGFR–PI3K–AKT–MAPK, integrin–FAK–Rho, and EMT programs (Fig. 13, Fig. 14 and Fig. 15) [94]. Collectively, these pathways regulate proliferation, fibroblast activation, ECM deposition, angiogenesis, and immune checkpoint induction. Mechanistically, EXT1 elongates HS chains that form heparan sulfate proteoglycan (HSPG), which act as co-receptors for FGF, VEGF, TGF-β, and LPA. By clustering receptors and stabilizing ligand binding, EXT1 amplifies oncogenic cascades, positioning it as a signal amplifier at the glycosylation–inflammation axis [70,95]. To explore therapeutic avenues, we further tested whether EXT1 could be targeted pharmacologically. Docking and molecular dynamics simulations revealed that 17-AAG, an HSP90 inhibitor, binds EXT1 with favorable energy and stable hydrogen bonds (Fig. 16). RMSD and RMSF profiles confirmed structural stabilization, suggesting possible allosteric inhibition [96,97]. While preliminary, these results highlight EXT1’s druggability and encourage the development of dedicated inhibitors or antibodies. Finally, to validate our computational and molecular insights at the protein level, we performed immunohistochemistry. EXT1 was progressively upregulated in advanced-stage PAAD and LUAD, with strong localization in epithelial and stromal compartments (Fig. 17A–D). These findings confirmed that EXT1 expression increases with tumor stage and validate its clinical significance.

Taken together, our results establish EXT1 as a mechanistic hub at the intersection of glycosylation and inflammation. Unlike glycan-binding proteins that act as interpreters of existing glycan structures, EXT1 directly initiates the biosynthesis of HS proteoglycans, thereby shaping the biochemical and structural landscape of the TME. By coupling growth factor signaling to stromal activation and immune checkpoint engagement, EXT1 contributes to fibrosis in PAAD, angiogenesis in LUAD, and immune evasion across cancers. Its promoter hypomethylation provides an additional layer of regulation, supporting its use as an epigenetic biomarker. Furthermore, as an enzyme with a defined catalytic activity, EXT1 is inherently druggable, making it an attractive candidate for therapeutic targeting. Thus, by integrating transcriptomic, epigenomic, single-cell, pathway enrichment, molecular modeling, and IHC validation, our study highlights EXT1 as a previously underexplored glycosyltransferase with broad clinical relevance. These findings extend the paradigm of cancer glycosylation biology beyond glycan-binding proteins, demonstrating that enzymatic regulators of glycosylation actively construct oncogenic signaling networks and represent actionable vulnerabilities in inflammation-driven malignancies. 

## 5 Conclusion

**Figure 18:**
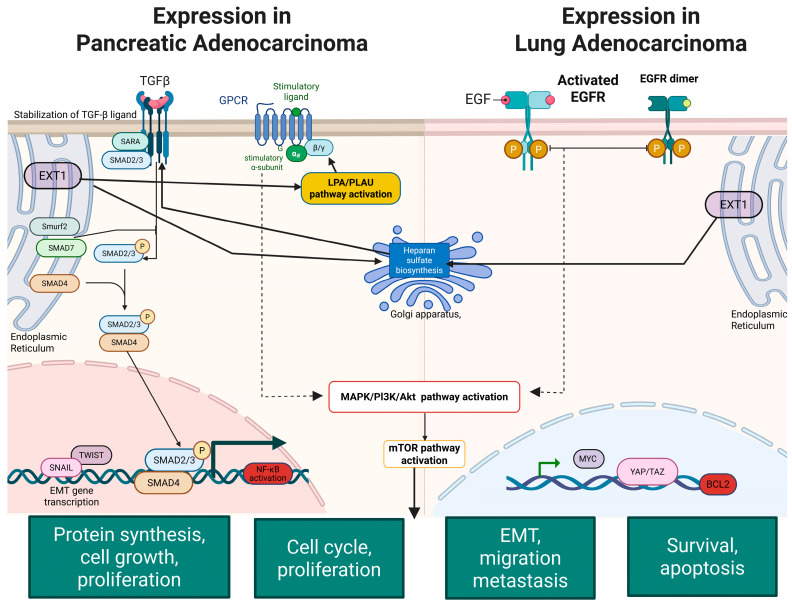
EXT1-driven oncogenic mechanisms in LUAD (left) and PAAD (right). EXT1 expression enhances HS biosynthesis in the ER and Golgi, promoting growth factor signaling in both cancers. In LUAD (left), stabilized ligands such as EGF activate the EGFR–PI3K–AKT–mTOR axis, driving proliferation and survival, while LPA–RhoA signaling remodels the cytoskeleton and induces nuclear YAP/TAZ activity to support motility. In PAAD (right), EXT1 stabilizes TGF-β, which activates SMAD-dependent EMT via nuclear transcription, while LPA/integrin signaling activates PI3K–AKT and mTOR pathways to promote cell proliferation. Concurrent cytokine signaling (IL-6, IL-17) induces NF-κB and STAT3 activation, supporting stromal activation and immune suppression. This figure was created by the authors using a licensed BioRender account (m654112009@tmu.edu.tw).

In this study, we examined the intersection of inflammation, glycosylation, and tumor biology and identified EXT1 as a key molecular integrator with prognostic significance across several cancers. Using a validated inflammation-related gene signature, we showed that EXT1 can stratify patient prognosis and is linked to more aggressive tumor phenotypes. Multi-omics analyses revealed consistent upregulation of EXT1 in tumors with immunosuppressive microenvironments and poor outcomes, and its associations with immune exclusion, pro-inflammatory signaling, stromal activation, and genomic instability point to a complex role in shaping tumor progression. Pathway enrichment and molecular modeling suggest that EXT1 may influence multiple oncogenic pathways and could potentially be targeted pharmacologically, while protein-level validation supports its value as a diagnostic and prognostic marker. Nonetheless, our conclusions are based largely on computational and IHC analyses, and direct *in vitro* and *in vivo* experiments will be essential to confirm its mechanistic role and therapeutic potential. Taken together, our findings position EXT1 as a promising biomarker and a candidate mediator of glycosylation–inflammation crosstalk, offering new insights into tumor immune evasion and resistance to therapy (Fig. 18).

## 6 Limitations

Despite the comprehensive multi-omics and integrative analytical approach, several limitations should be acknowledged. First, although extensive computational analyses and immunohistochemical validation were performed, the study lacks direct functional validation. No in vitro or in vivo assays were conducted to experimentally confirm the mechanistic roles of EXT1 in immune modulation, glycosylation processes, or extracellular matrix remodeling. Future investigations using CRISPR-mediated gene editing, functional glycoproteomics, and relevant animal models will be necessary to validate these predicted biological functions. Second, the glycosylation-related analyses relied primarily on bulk and single-cell transcriptomic datasets. While RNA-seq data provides valuable insights into gene expression patterns, they cannot directly capture post-translational glycosylation events. Because glycosylation is a dynamic biochemical modification, transcriptomic signals alone cannot fully reflect glycan structure, composition, or enzymatic activity. Dedicated glycoproteomic and metabolomic profiling will therefore be required to validate and extend the transcriptome-based predictions of EXT1-associated glycosylation processes. Another limitation relates to the resolution and scale of available single-cell datasets. Although scRNA-seq analysis provided important insights into cell-type-specific expression patterns of EXT1, the number of patient samples in currently available datasets remains relatively limited, which may affect the generalizability of the findings. In addition, standard scRNA-seq approaches lack spatial context within the tumor microenvironment. Future studies incorporating larger multi-cohort single-cell datasets together with spatial transcriptomics technologies will be important to better characterize the microenvironmental dynamics associated with EXT1. Furthermore, our pan-cancer analyses revealed context-dependent associations of EXT1 with tumor mutation burden, microsatellite instability, and immune phenotypes across different tumor types. The biological mechanisms underlying these divergent roles remain unclear and warrant further investigation using patient-derived organoids, co-culture systems, and in vivo perturbation models. In addition, although we developed a nomogram integrating EXT1 expression with an inflammation-related risk score that demonstrated favorable calibration and ROC performance, the predictive value of this model remains to be validated in prospective clinical cohorts, particularly in the context of immunotherapy response prediction. Finally, while molecular docking and dynamics simulations suggested that 17-AAG may interact with EXT1, this compound is primarily known as a heat shock protein inhibitor with broad pleiotropic effects. Therefore, the therapeutic implications remain preliminary. The development and experimental testing of specific EXT1 inhibitors or functional blocking antibodies will be required before clinical translation can be realistically considered.

## Data Availability

All data involved in this study are available from the corresponding author on request. Molecular Signatures Database (MSigDB, https://www.gsea-sigdb.org/gsea/msigdb/human/collections.jsp) for analysis in this.

## Supplementary Materials

The supplementary material is available online at https://www.techscience.com/doi/10.32604/or.2026.070445/s1.

## Abbreviations

AUC	Area under the curve
CAF	Cancer-associated fibroblast
CI	Confidence interval
COAD	Colorectal adenocarcinoma
DAB	3,3′-diaminobenzidine
DSS	Disease-specific survival
ECM	Extracellular matrix
EMT	Epithelial–mesenchymal transition
EXT1	Exostosin-1
FGF	Fibroblast growth factor
GBMLGG	Glioblastoma and lower-grade glioma
HNSC	Head and neck squamous cell carcinoma
HR	Hazard ratio
HS	Heparan sulfate
HSPG	Heparan sulfate proteoglycan
IHC	Immunohistochemistry
KIRC	Kidney renal clear cell carcinoma
KIRP	Kidney renal papillary cell carcinoma
LIHC	Liver hepatocellular carcinoma
LUAD	Lung adenocarcinoma
LUSC	Lung squamous cell carcinoma
MESO	Mesothelioma
MSI	Microsatellite instability
MSigDB	Molecular Signatures Database
NK cell	Natural killer cell
OS	Overall survival
PAAD	Pancreatic adenocarcinoma
PFI	Progression-free interval
PSCs	Pancreatic stellate cells
ROC	Receiver operating characteristic
scRNA-seq	Single-cell RNA sequencing
TCGA	The Cancer Genome Atlas
TCR	T cell receptor
TGF-β	Transforming growth factor-beta
TILs	Tumor-infiltrating lymphocytes
TMB	Tumor mutational burden
TME	Tumor microenvironment
TNM	Tumor–Node–Metastasis staging
VEGF	Vascular endothelial growth factor
