# Supplementary material for: Multi-Omics and Single-Cell Dissection Reveals EXT1 as a Glycosylation-Linked Therapeutic Target in Cancer
Source: Oncol Res. 2026 May 21;34(6):19. doi: 10.32604/or.2026.070445 (PMC13223188; doi:10.32604/or.2026.070445)
Supplement: Supplementary file 1 [file OncolRes-34-70445-s001.zip › TSP_OR_70445-s001.docx]

**Supplementary Materials**


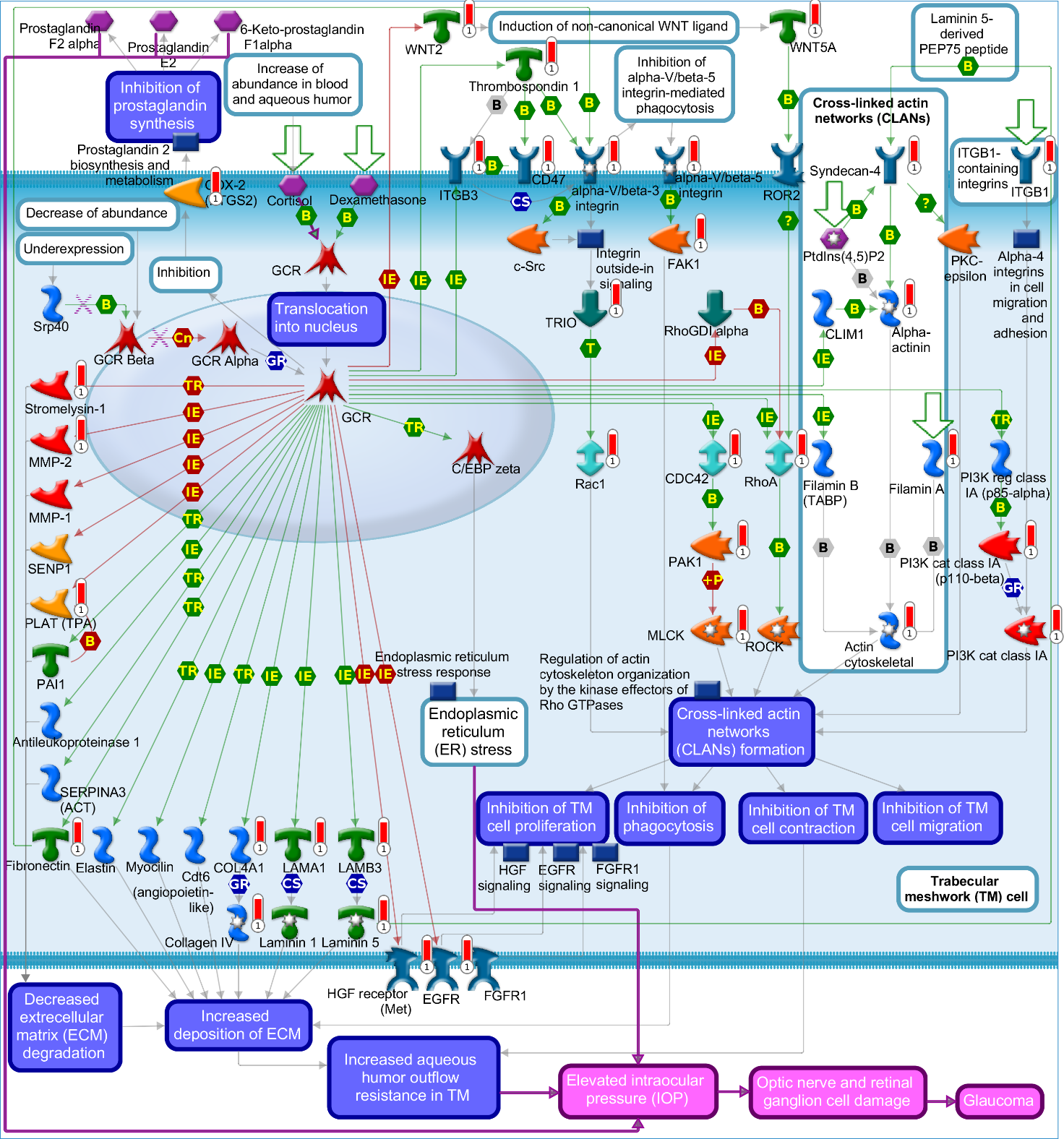


**Supplementary Figure S1.** **MetaCore pathway analysis of EXT1 co-expressed genes in PAAD patients from TCGA.** The “Glucocorticoid-induced elevation of intraocular pressure as glaucoma risk factor” is highlighted, with symbols representing proteins and arrows indicating protein interactions (green for activation and red for inhibition). Thermometer-like histograms visually represent microarray gene expressions, with blue indicating downregulation and red indicating upregulation**.**


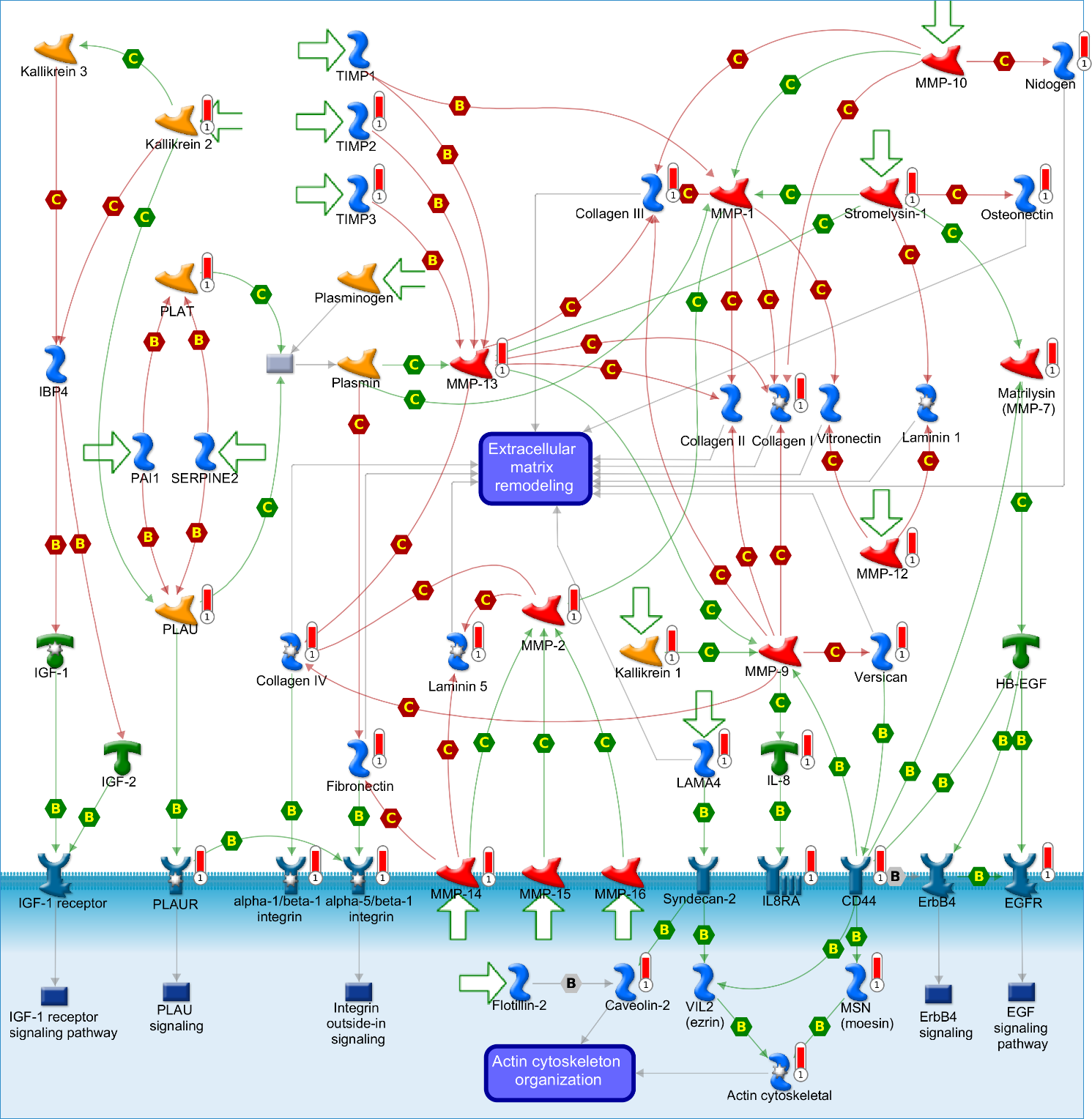


**Supplementary Figure S2.** **MetaCore pathway analysis of EXT1 co-expressed genes in PAAD patients from TCGA.** The “Cell adhesion_ECM remodeling” is highlighted, with symbols representing proteins and arrows indicating protein interactions (green for activation and red for inhibition). Thermometer-like histograms visually represent microarray gene expressions, with blue indicating downregulation and red indicating upregulation**.**


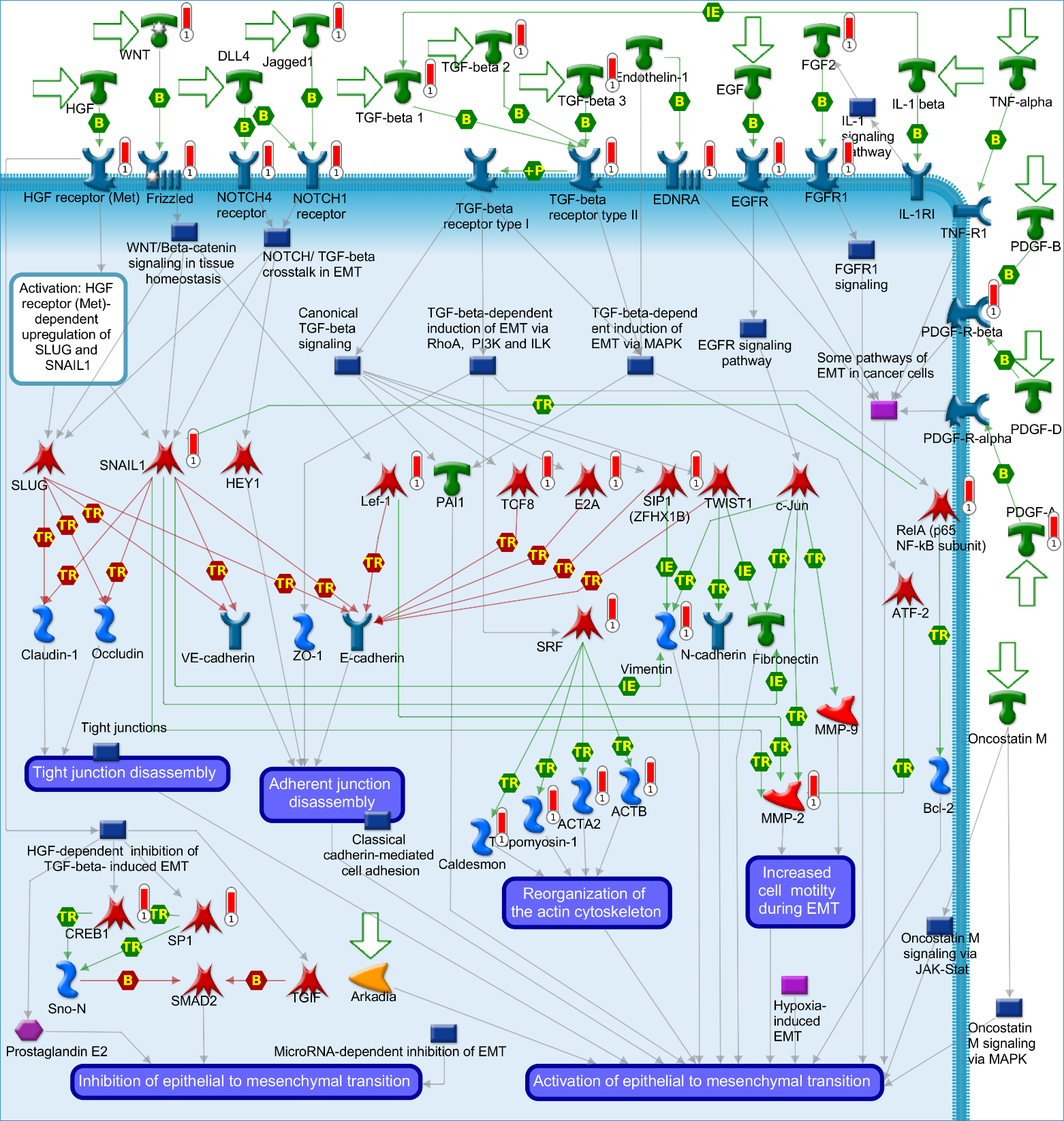


**Supplementary Figure S3.** **MetaCore pathway analysis of EXT1 co-expressed genes in LUAD patients.** The “Development_Regulation of epithelial-to-mesenchymal transition (EMT)” is highlighted, with symbols representing proteins and arrows indicating protein interactions (green for activation and red for inhibition). Thermometer-like histograms visually represent microarray gene expressions, with blue indicating downregulation and red indicating upregulation**.**


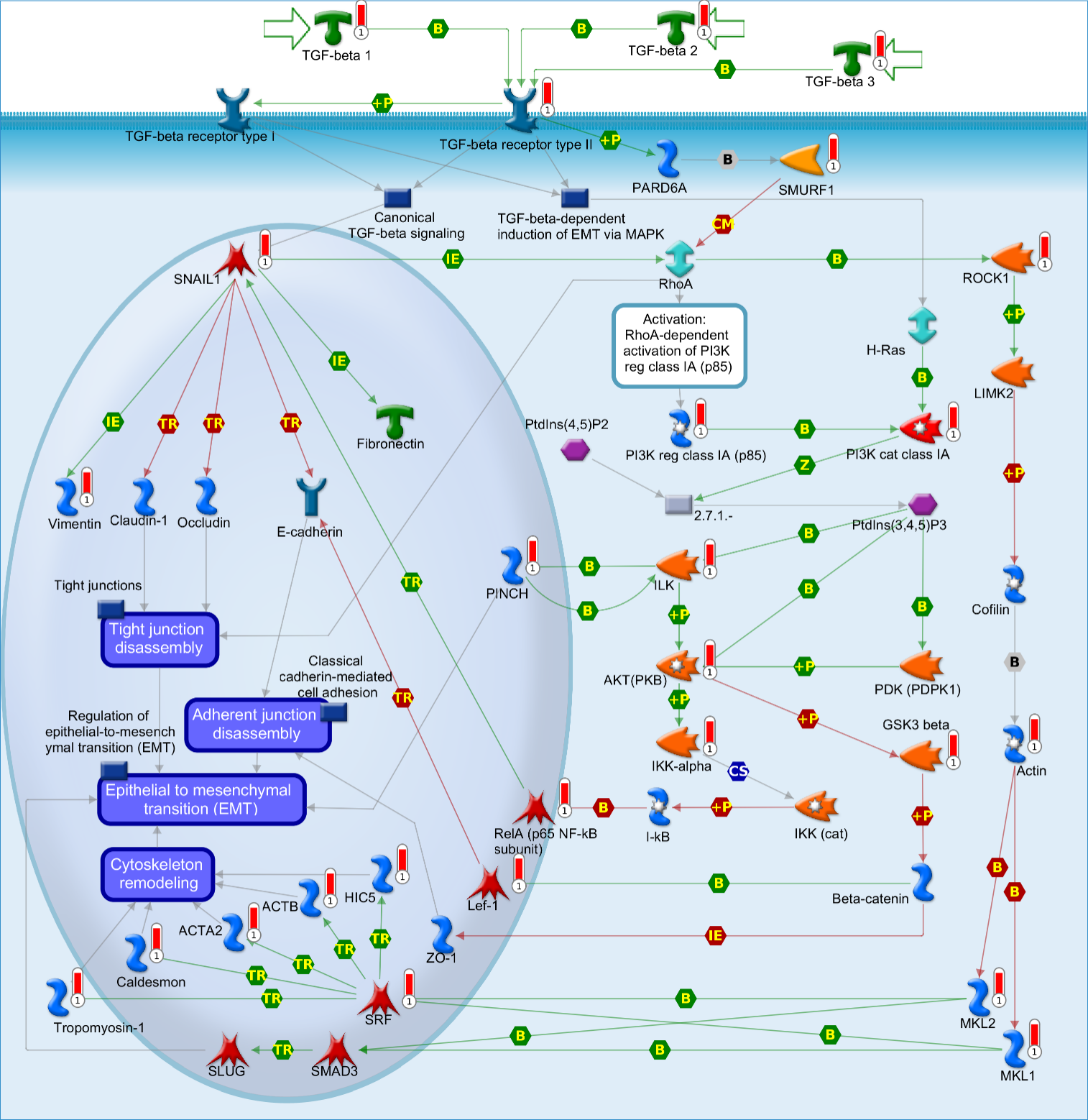


**Supplementary Figure S4**. **MetaCore pathway analysis of EXT1 co-expressed genes in LUAD patients.** The “Development_TGF-beta-dependent induction of EMT *via* RhoA, PI3K and ILK” is highlighted, with symbols representing proteins and arrows indicating protein interactions (green for activation and red for inhibition). Thermometer-like histograms visually represent microarray gene expressions, with blue indicating downregulation and red indicating upregulation**.**

**Supplementary Table S1:** **Pathway analysis of genes co-expressed with EXT1 co-expressed genes in PAAD TCGA databases using the MetaCore database (with *p*-value < 0.05 set as the cutoff value).**

| # | Maps | *p*Value | Network Objects from Active Data |
| --- | --- | --- | --- |
| 1 | Cell adhesion_PLAU signaling | 5.83E-21 | Casein kinase II, alpha chains, STAT3, MEK1(MAP2K1), FPRL1, RhoA, alpha-3/beta-1 integrin, c-IAP2, Nucleolin, PI3K cat class IA, LAMC2, Survivin, JAK1, c-IAP1, alpha-V/beta-1 integrin, alpha-V/beta-3 integrin, EGFR, G-protein alpha-i family, F-Actin cytoskeleton, MRLC, ERK1/2, FAK1, PLAUR (uPAR), CDC42, alpha-5/beta-1 integrin, G-protein alpha-i3, NF-kB, PAK1, MYLK1, sUPAR, alpha-V/beta-5 integrin, PLAU (UPA), MLCK, FPR, STAT1, Rac1, PDGF-R-beta |
| 2 | Glucocorticoid-induced elevation of intraocular pressure as glaucoma risk factor | 1.32E-18 | TRIO, COX-2 (PTGS2), RhoA, ITGB1, Laminin 5, PI3K cat class IA, WNT5A, COL4A1, PLAT (TPA), PI3K cat class IA (p110-beta), ITGB3, alpha-V/beta-3 integrin, EGFR, WNT2, MMP-2, FAK1, Syndecan-4, CDC42, Actin cytoskeletal, Alpha-actinin, Filamin A, PAK1, LAMB3, Collagen IV, Thrombospondin 1, HGF receptor (Met), Fibronectin, Stromelysin-1, alpha-V/beta-5 integrin, LAMA1, MLCK, CD47, Rac1 |
| 3 | Cell adhesion_ECM remodeling | 1.56E-18 | Laminin 5, MMP-13, TIMP3, CD44, Caveolin-2, PLAT (TPA), MSN (moesin), IL-8, EGFR, Kallikrein 2, MMP-14, MMP-2, Versican, MMP-12, Matrilysin (MMP-7), PLAUR (uPAR), alpha-5/beta-1 integrin, Actin cytoskeletal, alpha-1/beta-1 integrin, Collagen IV, TIMP2, IL8RA, Fibronectin, Kallikrein 1, Nidogen, Osteonectin, Stromelysin-1, LAMA4, PLAU (UPA), Collagen I, Collagen III |
| 4 | Cell adhesion_Integrin-mediated cell adhesion and migration | 2.73E-17 | RhoA, alpha-3/beta-1 integrin, ITGB1, Laminin 5, VCAM1, PARD3, PKC, alpha-6/beta-1 integrin, ICAM1, Vinculin, PKC-lambda/iota, PINCH, ITGB3, alpha-V/beta-3 integrin, 14-3-3 zeta/delta, F-Actin cytoskeleton, LARG, FAK1, alpha-2/beta-1 integrin, CDC42, alpha-5/beta-1 integrin, Actin cytoskeletal, Alpha-parvin, Alpha-actinin, CRK, alpha-1/beta-1 integrin, PAK1, Collagen IV, Fibronectin, Collagen I, Rac1, Collagen III |
| 5 | Protein folding and maturation_Amyloid precursor protein processing (schema) | 9.13E-17 | APP-C31, APP-CTF delta-short, APP-CTF delta-long, betaAPPs, ADAM9, deltaAPPs-80kD, Caspase-6, APP-P3, alphaAPPs, etaAPP beta, APP-CTF theta, Caspase-3, APP-C99, APP-C83 (CTF), etaAPP alpha, APP-NCas, Amyloid beta 40, APP, APP-CTF eta, thetaAPPs, Amyloid beta, ADAM10, etaAPPs, ADAM17, APP-C59 (AICD), deltaAPPs-130kD, APP-Jcasp, Amyloid beta 42 |
| 6 | TGF-beta signaling *via* kinase cascades in breast cancer | 1.47E-15 | COX-2 (PTGS2), MEK1(MAP2K1), p38alpha (MAPK14), TGF-beta 2, ITGB1, Amphiregulin, MMP-13, Survivin, ITGAV, NF-kB1 (p50), ADAM12, ATF-2, ITGB3, IL-8, alpha-V/beta-3 integrin, EGFR, TRAF6, ERK1/2, MMP-2, FAK1, JNK(MAPK8-10), TGF-alpha, TAB2, TWIST1, TIMP2, ADAM17, p38 MAPK, Rac1, TGF-beta receptor type I |
| 7 | Adhesion of small cell lung cancer (SCLC) cells in tumor progression | 6.33E-15 | RhoA, alpha-3/beta-1 integrin, E-cadherin, ITGB1, VCAM1, alpha-6/beta-1 integrin, ICAM1, Tenascin-C, alpha-V/beta-1 integrin, MMP-14, MMP-2, FAK1, alpha-2/beta-1 integrin, CDC42, alpha-5/beta-1 integrin, PTHrP, CD9, Collagen IV, Fibronectin, Stromelysin-1, CXCR4, Rac1 |
| 8 | TGF-beta 1-induced transactivation of membrane receptors signaling in hepatocellular carcinoma (HCC) | 1.28E-14 | alpha-3/beta-1 integrin, E-cadherin, ITGB1, Laminin 5, PI3K cat class IA, GSK3 beta, ITGA2, alpha-6/beta-1 integrin, SLUG, FAK1, alpha-2/beta-1 integrin, Beta-catenin, Cyclin A, alpha-5/beta-1 integrin, TGF-beta, CRK, ITGA6, PAK1, PDGF receptor, ITGA5, Fibronectin, Actin, Rac1, TGF-beta receptor type I, PDGF-R-beta, CDK2 |
| 9 | IGF family, invasion and metastasis in colorectal cancer | 1.84E-14 | RhoA, alpha-3/beta-1 integrin, E-cadherin, ITGB1, PI3K cat class IA, ITGAV, PLAT (TPA), IRS-1, Alpha-1 catenin, Beta-catenin, PLAUR (uPAR), alpha-V/beta-6 integrin, alpha-1/beta-1 integrin, Collagen IV, Fibronectin, alpha-V/beta-5 integrin, p38 MAPK, ERK1 (MAPK3), PLAU (UPA), Collagen I, Rac1 |
| 10 | Development_Positive regulation of WNT/Beta-catenin signaling in the cytoplasm | 2.45E-14 | Casein kinase II, alpha chains, COX-2 (PTGS2), PPP2R2A, HECTD1, ITGB1, BIG1, SIAH2, USP9X, IRS-1, PR130, WNT, PP1-cat, 14-3-3 zeta/delta, Alpha-1 catenin, FAK1, 14-3-3, TGT, GSK3 alpha/beta, Beta-catenin, PKA-reg type II (cAMP-dependent), TBLR1, JNK(MAPK8-10), TGIF, PP2A catalytic, ERK2 (MAPK1), MITF, Frizzled, SET7, Rac1, YAP1 (YAp65), CDK1 (p34), DACT1 |
| 11 | Chemotaxis_Lysophosphatidic acid signaling *via* GPCRs | 1.36E-13 | RhoA, E-cadherin, LPAR4, G-protein alpha-12 family, GSK3 beta, Caspase-7, PAK, PKC, alpha-6/beta-1 integrin, Vinculin, PI3K cat class IA (p110-beta), ATF-2, DIA1, IL-8, HAS2, alpha-V/beta-3 integrin, EGFR, G-protein alpha-i family, TRAF6, F-Actin cytoskeleton, Caspase-3, LARG, FasR(CD95), ERK1/2, PRK1, FAK1, CREB1, Beta-catenin, MEK1/2, G-protein beta/gamma, Rho GTPase, CDC42, Actin cytoskeletal, TAZ, CRK, JNK(MAPK8-10), G-protein alpha-q/11, ADAM17, p38 MAPK, Rac1, YAP1 (YAp65), G-protein gamma 12 |
| 12 | TGF-beta-induced fibroblast/ myofibroblast migration and extracellular matrix production in asthmatic airways | 3.69E-13 | COL1A1, ITGA1, TGF-beta 2, ITGB1, PI3K cat class IA, COL4A1, MMP-13, TIMP3, ITGA2, ITGA3, Tenascin-C, HAS2, ERK1/2, MMP-2, JNK(MAPK8-10), COL1A2, COL5A1, Collagen IV, TIMP2, ITGA5, Fibronectin, Stromelysin-1, p38 MAPK, Collagen I, TGF-beta receptor type I, Collagen III, Thrombospondin 2 |
| 13 | Development_Negative regulation of STK3/4 (Hippo) pathway and positive regulation of YAP/TAZ function | 9.79E-13 | MLCP (cat), RhoA, WW45, G-protein alpha-12 family, PARD3, SIAH2, CD44, STK3, ASPP1, EGFR, Itch, ASPP2, LARG, ERK1/2, LATS2, Mol1b, TAZ, JNK(MAPK8-10), RASSF6, PAR1, RhoGAP5, G-protein alpha-q/11, Actin, ERK1 (MAPK3), NEDD4, YAP1 (YAp65), ZO-2 |
| 14 | Effect of H. pylori infection on gastric epithelial cells motility | 1.17E-12 | E-cadherin, ITGB1, Vinculin, Lyn, ZO-1, Connexin 43, ERK1/2, FAK1, Occludin, Beta-catenin, alpha-5/beta-1 integrin, Actin cytoskeletal, Alpha-actinin, CRK, JNK(MAPK8-10), PAK1, JAM1, p120-catenin, Claudin-1, Actin, Alpha-catenin, Rac1 |
| 15 | Cytoskeleton remodeling_Integrin outside-in signaling | 6.97E-12 | TRIO, MEK1(MAP2K1), alpha-3/beta-1 integrin, alpha-11/beta-1 integrin, GSK3 beta, Vinculin, PINCH, alpha-V/beta-3 integrin, ERK1/2, FAK1, alpha-2/beta-1 integrin, Beta-catenin, alpha-5/beta-1 integrin, Actin cytoskeletal, Alpha-parvin, Alpha-actinin, Filamin A, PAK1, Collagen IV, Fibronectin, Collagen I, Rac1, WIRE |
| 16 | Development_VEGF signaling *via* VEGFR2 - generic cascades | 1.67E-11 | COX-2 (PTGS2), SPHK1, MEK1(MAP2K1), RhoA, I-kB, COX-1 (PTGS1), PI3K cat class IA, GSK3 beta, Calcineurin A (catalytic), PKC, NCK1, Vinculin, alpha-V/beta-3 integrin, PAK2, ERK1/2, FAK1, CREB1, Neurofibromin, Beta-catenin, PLAUR (uPAR), CDC42, Actin cytoskeletal, Fyn, PAK1, ERK2 (MAPK1), HSP90, Calcineurin B (regulatory), p38 MAPK, ERK1 (MAPK3), PLAU (UPA), MLCK, Rac1 |
| 17 | Role of Tissue factor in cancer independent of coagulation protease signaling | 1.79E-11 | MEK1(MAP2K1), alpha-3/beta-1 integrin, Laminin 5, PI3K cat class IA (p110-alpha), alpha-6/beta-1 integrin, ATF-2, Lyn, ERK1/2, FAK1, YES, CDC42, Actin cytoskeletal, Filamin A, PAK1, ERK2 (MAPK1), Thrombospondin 1, p38 MAPK, ERK1 (MAPK3), Rac1 |
| 18 | FAK1 signaling in melanoma | 2.1E-11 | RhoA, ITGB1, B-Raf, Syntenin 1, ITGB3, alpha-V/beta-3 integrin, N-Ras, ERK1/2, MMP-2, FAK1, MEK1/2, alpha-5/beta-1 integrin, Actin cytoskeletal, CRK, NF-kB, RhoC, ITGA5, Fibronectin, PLAU (UPA), Rac1 |
| 19 | Immune response_Plasmin signaling | 3.08E-11 | STAT3, COX-2 (PTGS2), MEK1(MAP2K1), p38alpha (MAPK14), CCL20, IL-1 alpha, PKC, NF-kB1 (p50), JAK1, ICAM1, c-Rel (NF-kB subunit), PLAT (TPA), ATF-2, ITGB3, Annexin II, Histone H2B, Caspase-3, ERK1/2, Protein kinase G, MEK1/2, PLAUR (uPAR), ENO1, PAR1, NF-kB, p38 MAPK, PLAU (UPA), STAT1 |
| 20 | CHDI_Correlations from Discovery data_Causal network (positive) | 3.46E-11 | STAT3, alpha-3/beta-1 integrin, JAK1, Vinculin, MMP-2, FAK1, alpha-2/beta-1 integrin, CDC42, alpha-5/beta-1 integrin, Actin cytoskeletal, IFN-alpha/beta receptor, Collagen IV, Thrombospondin 1, Actin, LAMA3 (Epiligrin), CD47, STAT1, Rac1, Collagen III |
| 21 | Role of alpha-V/ beta-6 integrin in colorectal cancer | 6.52E-11 | PKC, ITGAV, LTBP1, ETS1, Caspase-3, ERK1/2, MMP-2, MEK1/2, alpha-V/beta-6 integrin, ERK2 (MAPK1), Collagen IV, Fibronectin, ITGB6, PLAU (UPA), Collagen I |
| 22 | Cell adhesion_Endothelial cell contacts by non-junctional mechanisms | 1.59E-10 | alpha-3/beta-1 integrin, alpha-6/beta-1 integrin, alpha-V/beta-3 integrin, F-Actin cytoskeleton, alpha-2/beta-1 integrin, Beta-catenin, alpha-5/beta-1 integrin, Alpha-actinin, alpha-1/beta-1 integrin, Collagen IV, Fibronectin, p120-catenin, alpha-V/beta-5 integrin, Alpha-catenin, Collagen I |
| 23 | Development_TGF-beta-dependent induction of EMT *via* MAPK | 1.84E-10 | MEK1(MAP2K1), p38alpha (MAPK14), E-cadherin, TGF-beta 2, ITGB1, NOX4, ATF-2, ITGB3, alpha-V/beta-1 integrin, ERK1/2, MMP-2, FAK1, Occludin, TGF-beta, JNK(MAPK8-10), DAB2, Fibronectin, Claudin-1, p38 MAPK, Rac1, TGF-beta receptor type I |
| 24 | Induction of fibrosis in systemic sclerosis | 1.88E-10 | COL1A1, STAT3, IL-1 alpha, IL13RA1, JAK1, Tenascin-C, KLF5, WNT, TLR4, ERK1/2, DKK1, Beta-catenin, MEK1/2, ACTA2, SMAD1, JNK(MAPK8-10), COL1A2, COL5A1, PDGF receptor, Thrombospondin 1, Fibronectin, Stromelysin-1, Fra-2, Cathepsin V, alpha-V/beta-5 integrin, Frizzled, EDNRA, TGF-beta receptor type I, Collagen III |
| 25 | Extracellular matrix-regulated proliferation of airway smooth muscle cells in asthma | 2.02E-10 | COL1A1, PI3K cat class IA, ITGA3, ATF-2, EGFR, ERK1/2, alpha-2/beta-1 integrin, alpha-5/beta-1 integrin, JNK(MAPK8-10), Collagen IV, DAB2, Fibronectin, JNK1(MAPK8), p38 MAPK, LAMA1, Collagen I, TGF-beta receptor type I, Collagen III |
| 26 | TGF-beta 1-mediated induction of EMT in normal and asthmatic airway epithelium | 2.04E-10 | COL1A1, E-cadherin, CD44, Tenascin-C, ITGB3, ETS1, SLUG, ZO-1, MMP-2, Occludin, ACTA2, JNK(MAPK8-10), HMGA2, TWIST1, DAB2, Fibronectin, Jagged1, JNK1(MAPK8), p38 MAPK, TGF-beta receptor type I |
| 27 | FGF2 signaling in melanoma | 2.12E-10 | STAT3, RhoA, PI3K cat class IA, B-Raf, Syndecan-1, Heparanase 1, N-Ras, ERK1/2, MMP-2, Syndecan-4, YES, MEK1/2, CDC42, Collagen IV, Fibronectin, p38 MAPK, Collagen I, Rac1, CDK1 (p34) |
| 28 | Cytoskeleton remodeling_Regulation of actin cytoskeleton organization by the kinase effectors of Rho GTPases | 4.66E-10 | MLCP (cat), RhoA, PAK, Rac1-related, Cdc42 subfamily, Vinculin, MSN (moesin), ERM proteins, F-Actin cytoskeleton, Spectrin, MRLC, PRK1, RhoA-related, Caldesmon, CDC42, Actin cytoskeletal, Alpha-actinin, Filamin A, PAK1, RhoC, MyHC, MLCK, Rac1 |
| 29 | Role of cell adhesion molecules in progression of pancreatic cancer | 5.62E-10 | MEK1(MAP2K1), alpha-3/beta-1 integrin, E-cadherin, ITGB1, Caspase-7, alpha-6/beta-1 integrin, K-RAS, alpha-V/beta-3 integrin, Caspase-3, ERK1/2, FAK1, alpha-2/beta-1 integrin, Beta-catenin, alpha-5/beta-1 integrin, Collagen IV, ITGA5, Fibronectin, SIAT8B, Alpha-catenin, Collagen I |
| 30 | Development_Regulation of epithelial-to-mesenchymal transition (EMT) | 7.61E-10 | E-cadherin, TGF-beta 2, ATF-2, WNT, SLUG, EGFR, ZO-1, Sno-N, MMP-2, CREB1, Occludin, Caldesmon, ACTA2, Tropomyosin-1, TGIF, TWIST1, HGF receptor (Met), Fibronectin, Jagged1, Claudin-1, Frizzled, EDNRA, TGF-beta receptor type I, PDGF-R-beta |
| 31 | Transcription_HIF-1 targets | 7.87E-10 | TfR1, TGF-beta 2, GLUT1, Ceruloplasmin, Carbonic anhydrase XII, Lysyl oxidase, HXK2, HIF1A, HXK1, P4HA2, LOXL2, MMP-2, TGM2, Galectin-1, NOXA, PLAUR (uPAR), P4HA1, Cyclin G2, 5'-NTD, ENO1, Angiopoietin 2, LDHA, Thrombospondin 1, HGF receptor (Met), MSH6, PGK1, CXCR4, LRP1, AK3, IBP3 |
| 32 | Signal transduction_S1P2 receptor activation signaling | 1.02E-09 | STAT3, MLCP (cat), COX-2 (PTGS2), RhoA, G-protein alpha-12 family, PI3K cat class IA, GSK3 beta, VCAM1, Survivin, BMP receptor 2, RUNX2, ICAM1, G-protein alpha-i family, MRLC, LARG, FOXM1, ERK1/2, FAK1, CREB1, Beta-catenin, MEK1/2, ACTA2, G-protein beta/gamma, Actin cytoskeletal, G-protein alpha-q, SMAD1, JNK(MAPK8-10), NF-kB, Osteoprotegerin, p38 MAPK, YAP1 (YAp65), G-protein alpha-13 |
| 33 | Mechanisms of drug resistance in small cell lung cancer (SCLC) | 1.12E-09 | alpha-3/beta-1 integrin, ABCC1, ITGB1, Osteopontin, PI3K cat class IA, GSK3 beta, VCAM1, B-Raf, Survivin, alpha-6/beta-1 integrin, c-IAP1, alpha-V/beta-1 integrin, alpha-V/beta-3 integrin, Caspase-3, ERK1/2, FAK1, TOP2 alpha, alpha-2/beta-1 integrin, alpha-5/beta-1 integrin, CD9, Collagen IV, HSP70, Fibronectin, Rad51, HSPA1A |
| 34 | Signal transduction_S1P1 receptor signaling | 1.17E-09 | COX-2 (PTGS2), SPHK1, MEK1(MAP2K1), RhoA, PI3K cat class IA (p110-alpha), GSK3 beta, ICAM1, RANKL(TNFSF11), PI3K cat class IA (p110-beta), KLF5, cPLA2, alpha-V/beta-3 integrin, EGFR, G-protein alpha-i family, ZO-1, ERK1/2, MMP-14, FAK1, Beta-catenin, MEK1/2, G-protein beta/gamma, CDC42, Fyn, Alpha-actinin, CRK, JNK(MAPK8-10), G-protein alpha-i3, PAK1, Osteoprotegerin, p38 MAPK, Alpha-catenin, Rac1, YAP1 (YAp65), PDGF-R-beta |
| 35 | Development_Negative regulation of WNT/Beta-catenin signaling in the cytoplasm | 1.44E-09 | WWP1, E-cadherin, HECTD1, WNT5A, STK3, WNT, PP1-cat, Amer1, Presenilin 1, Itch, Alpha-1 catenin, GSK3 alpha/beta, LATS2, Beta-catenin, G-protein beta/gamma, TAZ, PP2A catalytic, PR72, DAB2, JNK1(MAPK8), Frizzled, YAP1/TAZ, Rac1, YAP1 (YAp65), G-protein alpha-13, DACT1, CDK6 |
| 36 | Role of endothelial and immune cells in systemic sclerosis | 1.52E-09 | COL1A1, VCAM1, GCP2, ICAM1, IL-8, TLR4, Neuropilin-1, FasR(CD95), ENA-78, TEF-1, MMP-12, ACTA2, PLAUR (uPAR), SMAD1, COL1A2, Fibronectin, Fra-2, Cathepsin V, p38 MAPK, Cathepsin B, EDNRA, TGF-beta receptor type I, YAP1 (YAp65) |
| 37 | Signal transduction_Thrombospondin 1 signaling | 1.58E-09 | alpha-3/beta-1 integrin, ITGB1, Rap1, PI3K cat class IA, Protein kinase G1, alpha-6/beta-1 integrin, ITGB3, alpha-V/beta-3 integrin, EGFR, G-protein alpha-i family, ERK1/2, FAK1, alpha-2/beta-1 integrin, MEK1/2, Fyn, PKA-reg (cAMP-dependent), JNK(MAPK8-10), DR4(TNFRSF10A), HSP90, Thrombospondin 1, JNK1(MAPK8), p38 MAPK, CD47, CDK2, CDK6 |
| 38 | HGF signaling in melanoma | 1.93E-09 | MEK1(MAP2K1), E-cadherin, PI3K cat class IA, GSK3 beta, B-Raf, CD44, HIF1A, ATF-2, SLUG, N-Ras, ERK1/2, Beta-catenin, JNK(MAPK8-10), TWIST1, HGF receptor (Met), Fibronectin, p38 MAPK, MITF |
| 39 | Immune response_Antigen presentation by MHC class I: cross-presentation | 2.38E-09 | SEC22B, Rab-6, Rab-27A, HYOU1, Rab-7, IRAP, UFO, MSR1, cPLA2, TLR4, Rab-10, OLR1, Calreticulin, Connexin 43, ARF6, LY75, HSP90 alpha, FCGR3A, Rab-32, p67-phox, HSP60, Fc gamma RII alpha, HSP90, HSP70, Rab8B, TLR3, SNAP-23, LRP1, Cathepsin B, HSPA1A |
| 40 | Cell cycle_Influence of Ras and Rho proteins on G1/S Transition | 2.71E-09 | STAT3, MLCP (cat), MEK1(MAP2K1), RhoA, PI3K cat class IA, GSK3 beta, Cyclin A2, ATF-2, DIA1, MRLC, ERK1/2, FAK1, CDC42, alpha-5/beta-1 integrin, PAK1, RalA, JNK1(MAPK8), MLCK, Rac1, CDK2, CDK6 |
| 41 | Inhibition of remyelination in multiple sclerosis: regulation of cytoskeleton proteins | 2.76E-09 | MLCP (cat), Tubulin beta, RhoA, MARCKS, Tubulin alpha, alpha-V/beta-1 integrin, MRLC, Tubulin beta 4, FAK1, chTOG, CDC42, TGF-beta, Actin cytoskeletal, Fyn, KLHL2, PAK1, Fibronectin, Rac1, Tubulin (in microtubules) |
| 42 | G protein-coupled receptors signaling in lung cancer | 2.89E-09 | STAT3, RhoA, I-kB, G-protein alpha-12 family, Amphiregulin, EDNRB, CD44, IL-8, cPLA2, alpha-V/beta-3 integrin, EGFR, G-protein alpha-i family, ERK1/2, MMP-2, G-protein beta/gamma, Galpha(i)-specific peptide GPCRs, PKA-reg (cAMP-dependent), BDKRB2, TGF-alpha, Galpha(q)-specific peptide GPCRs, G-protein alpha-q/11, IL8RA, ADAM17, CXCR4, EDNRA, IL8RB |
| 43 | G-protein signaling_RhoA inhibition | 2.89E-09 | RhoA, 14-3-3 beta/alpha, Rap1, Protein kinase G1, Cyclin B, SMURF1, 14-3-3 zeta/delta, FAK1, RhoE, 14-3-3 gamma, CDC42, TGF-beta, Fyn, PKA-reg (cAMP-dependent), RhoGAP5, PAK1, ERK2 (MAPK1), Aurora-A, ERK1 (MAPK3), BDNF, RAP-2B, SLK, Alpha-catenin, Rac1, CDK1 (p34), Tubulin (in microtubules) |
| 44 | IL-1 beta- and Endothelin-1-induced fibroblast/ myofibroblast migration and extracellular matrix production in asthmatic airways | 3.22E-09 | COL1A1, IL-1 alpha, COL4A1, EDNRB, TIMP3, HAS2, ERK1/2, MMP-2, Versican, COL1A2, NF-kB, Thrombospondin 1, Fibronectin, Stromelysin-1, EDNRA, Collagen I, PDGF-R-beta, Collagen III |
| 45 | Cell cycle_The metaphase checkpoint | 3.52E-09 | HZwint-1, ZW10, Survivin, Nek2A, CENP-A, MAD2a, BUBR1, INCENP, HEC, CENP-E, BUB1, Aurora-A, PLK1, CDCA1, CENP-F, Zwilch, AF15q14 |
| 46 | LKB1 signaling in lung cancer cells | 4.37E-09 | STAT3, COX-2 (PTGS2), p63, E-cadherin, TGF-beta 2, ITGB1, Lysyl oxidase, MO25, HIF1A, ERK1/2, FAK1, Beta-catenin, CDC42, NF-kB, PAK1, Collagen IV, Fibronectin, NFYA, Collagen I |
| 47 | HGF signaling in colorectal cancer | 4.52E-09 | COX-2 (PTGS2), E-cadherin, Laminin 5, PI3K cat class IA, GSK3 beta, LAMC2, CD44, EGFR, ERK1/2, MMP-2, FAK1, Beta-catenin, MEK1/2, PLAUR (uPAR), Actin cytoskeletal, JNK(MAPK8-10), LAMB3, TIMP2, Thrombospondin 1, HGF receptor (Met), ADAM10, LAMA3 (Epiligrin), Rac1 |
| 48 | Protein folding and maturation_Regulation of amyloid precursor protein processing | 5.57E-09 | ARL5B, Rap1, Rab-5A, GSK3 beta, betaAPPs, ADAM9, alphaAPPs, LRP10, CED-6, Tiam2, Presenilin 1, Calsyntenin-1, F-spondin, APP-C99, G-protein beta/gamma, G-protein alpha-q, JNK(MAPK8-10), Amyloid beta 40, PICALM, APP, DAB2, Amyloid beta, ADAM10, VPS35, SFK, ADAM17, BDNF, APP-C59 (AICD), LRP1, Rac1, Amyloid beta 42 |
| 49 | Signal transduction_FAK1 signaling | 5.82E-09 | TRIO, RhoA, ITGB1, PI3K cat class IA, ITGA2, ETS, EGFR, ERK1/2, MMP-14, MMP-2, FAK1, alpha-2/beta-1 integrin, MEK1/2, RAS, CRK, JNK(MAPK8-10), alpha-1/beta-1 integrin, PAK1, G-protein alpha-q/11, Collagen IV, PDGF receptor, Fibronectin, alpha-V/beta-5 integrin, Collagen I, Rac1 |
| 50 | Role of stellate cells in progression of pancreatic cancer | 6.34E-09 | COL1A1, MEK1(MAP2K1), PI3K cat class IA, MMP-13, IL-8, alpha-V/beta-3 integrin, EGFR, ERK1/2, MMP-2, FAK1, Galectin-1, ACTA2, alpha-5/beta-1 integrin, COL1A2, PDGF receptor, OSF-2, Fibronectin, Stromelysin-1, Collagen I, TGF-beta receptor type I, PDGF-R-beta, Collagen III |

**Supplementary Table S2: Pathway analysis of genes co-expressed with EXT1 co-expressed genes in LUAD databases using the MetaCore database (with *p*-value < 0.05 set as the cutoff value).**

| # | Maps | *p*Value | Network Objects from Active Data |
| --- | --- | --- | --- |
| 1 | Chemotaxis_Lysophosphatidic acid signaling *via* GPCRs | 5.07E-18 | MLCP (reg), p130CAS, ROCK, PI3K reg class IA (p85), cPKC (conventional), AP-1, GSK3 beta, PAK, PKC, Vinculin, PI3K cat class IA (p110-beta), Tcf(Lef), HAS2, EGFR, TRAF6, F-Actin cytoskeleton, LARG, ROCK1, FasR(CD95), MKL2, ERK1/2, Cyr61, PRK1, FAK1, ARHGEF1 (p115RhoGEF), CREB1, EGR1, G-protein beta/gamma, Rho GTPase, Actin cytoskeletal, N-CoR, TAZ, HDAC7, MKL1, CTGF, AKT(PKB), G-protein alpha-q/11, c-Src, PKC-epsilon, LIMK, ADAM17, PLC-epsilon, p38 MAPK, PLD2, SRF, Paxillin, YAP1 (YAp65), MEK4(MAP2K4) |
| 2 | Development_Regulation of epithelial-to-mesenchymal transition (EMT) | 4.79E-16 | TGF-beta 2, TGF-beta 1, RelA (p65 NF-kB subunit), NOTCH1 receptor, E2A, TGF-beta 3, WNT, TGF-beta receptor type II, EGFR, Lef-1, SIP1 (ZFHX1B), MMP-2, PDGF-A, CREB1, NOTCH4 receptor, Caldesmon, ACTA2, Tropomyosin-1, FGF2, SNAIL1, SP1, HGF receptor (Met), Jagged1, Vimentin, TCF8, SRF, Frizzled, EDNRA, FGFR1, ACTB, PDGF-R-beta |
| 3 | Development_TGF-beta-dependent induction of EMT *via* RhoA, PI3K and ILK | 8.34E-16 | TGF-beta 2, TGF-beta 1, PI3K reg class IA (p85), PI3K cat class IA, GSK3 beta, RelA (p65 NF-kB subunit), TGF-beta 3, PINCH, SMURF1, IKK-alpha, TGF-beta receptor type II, Lef-1, ROCK1, MKL2, Caldesmon, ACTA2, Tropomyosin-1, SNAIL1, MKL1, AKT(PKB), HIC5, Vimentin, Actin, SRF, ILK, ACTB |
| 4 | Development_Negative regulation of WNT/Beta-catenin signaling in the cytoplasm | 4.14E-15 | ELAVL1 (HuR), WWP1, PEG3, Prickle-1, WNT5A, NOTCH1 receptor, STK4, c-Cbl, SIAH1, PKC-alpha, STK3, Tcf(Lef), WNT, Amer1, beta-TrCP, Itch, GSK3 alpha/beta, Ankyrin-G, LATS2, CYLD, G-protein beta/gamma, WDR26, Skp2/TrCP/FBXW, TAZ, PR72, DAB2, Nucleoredoxin, NKD2, Dsh, RNF185, DACT3, Frizzled, YAP1/TAZ, YAP1 (YAp65) |
| 5 | Protein folding and maturation_Amyloid precursor protein processing (schema) | 1.26E-14 | APP-C31, APP-CTF delta-short, APP-CTF delta-long, betaAPPs, deltaAPPs-80kD, APP-P3, alphaAPPs, BACE1, MMP-24, etaAPP beta, APP-CTF theta, APP-C99, APP-C83 (CTF), etaAPP alpha, APP-NCas, Amyloid beta 40, APP, APP-CTF eta, thetaAPPs, Amyloid beta, etaAPPs, ADAM17, APP-C59 (AICD), deltaAPPs-130kD, APP-Jcasp, Amyloid beta 42 |
| 6 | Cytoskeleton remodeling_Regulation of actin cytoskeleton organization by the kinase effectors of Rho GTPases | 1.41E-14 | Talin, MLCP (reg), ROCK, DMPK, MRCKalpha, PAK, Cdc42 subfamily, RhoJ, Vinculin, MSN (moesin), ERM proteins, F-Actin cytoskeleton, MRLC, SLC9A1, PRK1, ARPC1B, Caldesmon, Actin cytoskeletal, Alpha-actinin, Filamin A, Alpha adducin, LIMK1, PIP5KI, MyHC, LIMK, MLCK, Paxillin, MRCK |
| 7 | Development_EGFR signaling | 3.06E-13 | NOTCH1 (NICD), p130CAS, PI3K reg class IA (p85), TrkA, PI3K cat class IA, AP-1, Amphiregulin, GSK3 beta, NOTCH1 receptor, PI3K reg class IA, GAB1, JAK1, c-Cbl, IKK-alpha, WNT, EGFR, RIPK1, ERK1/2, MMP-2, FAK1, EGR1, FGF2, NF-kB, CTGF, ERK2 (MAPK1), AKT(PKB), NIK(MAP3K14), NOTCH1 (NEXT), c-Src, p90Rsk, ILK, Paxillin |
| 8 | Signal transduction_ESR1 (membrane) and ESR2 (membrane) signaling | 1.19E-12 | SREBP1 precursor, CBP, PI3K reg class IA (p85), cPKC (conventional), PI3K cat class IA, Protein kinase G1, GSK3 beta, PI3K reg class IA (p85-alpha), PKC, PI3K reg class IA, ESR1 (nuclear), PKC-alpha, MSN (moesin), IRS-1, Tcf(Lef), EGFR, N-WASP, Adenylate cyclase, CaMK II alpha, ERK1/2, MMP-2, FAK1, CACNA1C, CREB1, ESR1 (membrane), EGR1, G-protein beta/gamma, Guanylate cyclase A (NPR1), AKAP5, GATA-1, AKT(PKB), Caveolin-1, FASN, c-Src, PKA-cat (cAMP-dependent), SRF, p90Rsk, PR (nuclear) |
| 9 | Signal transduction_RANKL-dependent osteoclast differentiation | 8.22E-12 | p38alpha (MAPK14), CBP, PI3K cat class IA, AP-1, GSK3 beta, RelA (p65 NF-kB subunit), Calcineurin A (catalytic), IFRD1, PI3K reg class IA, NF-kB1 (p50), JDP2, NF-AT1(NFATC2), Fra-1, TRAF6, Syndecan-4, CREB1, VAV-3, NF-kB, AKT(PKB), NIK(MAP3K14), FAM102A, MKK7 (MAP2K7), TAL1, ATP6V1C1, c-Src, p38 MAPK, MITF, NF-AT2(NFATC1), Cathepsin K, SH3BP-2 |
| 10 | Regulation of metabolism_GLP-1 signaling in beta cells | 8.41E-12 | Rap1, c-IAP2, NF-AT4(NFATC3), PI3K cat class IA, Calcineurin A (catalytic), PI3K reg class IA (p85-alpha), B-Raf, cAMP-GEFI, NF-AT1(NFATC2), PKA-cat alpha, cAMP-GEFII, IKK-alpha, TORC2, EGFR, Rab-3A, TCF7L2 (TCF4), Obestatin, ERK1/2, 14-3-3, CREB1, COUP-TFII, EGR1, G-protein beta/gamma, NF-kB, AKT(PKB), p300, NRF2, PDZ-GEF1, c-Src, NF-AT2(NFATC1), PKA-cat (cAMP-dependent), p90Rsk |
| 11 | Signal transduction_Calcium-mediated signaling | 1.03E-11 | MLCP (reg), ROCK, cPKC (conventional), Myocardin, MARK2, RelA (p65 NF-kB subunit), Calcineurin A (catalytic), PKC, MYH11, PKC-alpha, MALT1, TORC2, ERK1/2, 14-3-3, CABIN1, CREB1, EGR1, ACTA2, MEF2, NF-kB, HDAC5, AKT(PKB), p300, p38 MAPK, NF-AT2(NFATC1), SRF, MUNC13, MEK4(MAP2K4) |
| 12 | PI3K signaling in gastric cancer | 1.16E-11 | ELAVL1 (HuR), PI3K reg class IA (p85), PI3K cat class IA (p110-alpha), PI3K cat class IA, GSK3 beta, RelA (p65 NF-kB subunit), PI3K reg class IA (p85-alpha), PI3K reg class IA, PTEN, IRS-1, Neuregulin 1, IKK-alpha, HSP27, EGFR, Cyr61, FAK1, CBL-B, PRNP, AKT(PKB), G-protein alpha-q/11, NF-kB p50/p65, HGF receptor (Met), c-Src |
| 13 | Stellate cells activation and liver fibrosis | 3.02E-11 | Biglycan, TGF-beta 1, PI3K reg class IA (p85), PI3K cat class IA, GSK3 beta, PTCH1, CCL2, TRAF1, Tcf(Lef), TGF-beta receptor type II, TRAF6, KLF6, RIPK1, MMP-2, ACTA2, GLI-1, ERK2 (MAPK1), AKT(PKB), NIK(MAP3K14), SP1, PDGF receptor, NF-kB p50/p65, DAB2, Dsh, ERK1 (MAPK3), Frizzled, PDGF-R-beta |
| 14 | Cytoskeleton remodeling_PDGF signaling *via* calcium and Rho GTPases | 9.55E-11 | SLC31A1, p130CAS, ABL2, STIM1, PI3K reg class IA (p85), Schwannomin (NF2), PKC, F-Actin, Vinculin, PKC-alpha, N-WASP, FAK1, c-Abl, PDGF-A, CACNA1C, Guanylate cyclase A (NPR1), Actin cytoskeletal, Fyn, WASF subunit, PDGF receptor, ALPHA-PIX, Dynamin-2, c-Src, PKC-epsilon, PKA-cat (cAMP-dependent), WASF2, Paxillin, PDGF-R-beta, DOCK8 |
| 15 | Signal transduction_PDGF signaling *via* PI3K/AKT and NFkB pathways | 1.88E-10 | PI3K reg class IA (p85), PI3K cat class IA (p110-alpha), Myocardin, GSK3 beta, RelA (p65 NF-kB subunit), MYH11, PTEN, CCL2, PI3K cat class IA (p110-beta), ETS1, ERK1/2, MMP-2, c-Abl, PDGF-A, Transgelin, ACTA2, NF-kB, MKL1, AKT(PKB), SP1, PDGF receptor, NF-kB p50/p65, c-Src, SRF, Phox1 (PRRX1), PDGF-R-beta |
| 16 | Signal transduction_S1P2 receptor activation signaling | 2.22E-10 | MLCP (reg), ROCK, PI3K reg class IA (p85), PI3K cat class IA, AP-1, GSK3 beta, BMP receptor 2, MYH11, CCL2, beta-TrCP, MRLC, LARG, ROCK1, ERK1/2, FAK1, ARHGEF1 (p115RhoGEF), CREB1, Transgelin, EGR1, ACTA2, G-protein beta/gamma, Actin cytoskeletal, NF-kB, MKL1, AKT(PKB), LIMK1, S1P2 receptor, PPAR-gamma, p38 MAPK, SRF, TCF7 (TCF1), Paxillin, YAP1 (YAp65) |
| 17 | Signal transduction_Angiotensin II/ AGTR1 signaling *via* TGF-beta 1 and SMADs | 2.45E-10 | SREBP1 precursor, TGF-beta 1, AP-1, GSK3 beta, RelA (p65 NF-kB subunit), SREBP1 (nuclear), SMAD7, TGF-beta receptor type II, SMURF2, TRAF6, ERK1/2, NF-kB, CTGF, AKT(PKB), SP1, SPRY1, MKK7 (MAP2K7), Thrombospondin 1, FASN, p38 MAPK, MEK4(MAP2K4), CARD8 |
| 18 | Development_Positive regulation of WNT/Beta-catenin signaling in the cytoplasm | 2.74E-10 | Casein kinase II, alpha chains, TGF-beta 1, Trabid, BIG1, Miz-1, SIAH1, IRS-1, USP25, Tcf(Lef), PR130, WNT, USP7, 14-3-3 zeta/delta, FAK1, 14-3-3, GSK3 alpha/beta, ERK2 (MAPK1), AKT(PKB), Makorin-1, NKD2, Dsh, MITF, PKA-cat (cAMP-dependent), ILK, Frizzled, Beta-arrestin2, YAP1 (YAp65) |
| 19 | Development_NOTCH signaling inhibition | 2.88E-10 | Casein kinase II, alpha chains, NOTCH1 (NICD), NOTCH2 (2ICD), NOTCH4 (ICD4), KCTD10, ETO, GSK3 beta, FHL1 (SLIM1), NUMBL, NOTCH1 receptor, c-Cbl, BACE1, 14-3-3 zeta/delta, Itch, NOTCH1 precursor, J1ICD, NOTCH4 receptor, N-CoR, Skp2/TrCP/FBXW, SNAIL1, USP12, AKT(PKB), NOTCH2 receptor, Jagged1, c-Src, SHARP (SPEN), Nibrin, p38 MAPK, APP-C59 (AICD), NUMB, ILK |
| 20 | Stimulation of TGF-beta signaling in lung cancer | 2.94E-10 | SCUBE3, p38alpha (MAPK14), TGF-beta 2, TGF-beta 1, PI3K cat class IA (p110-alpha), RelA (p65 NF-kB subunit), PI3K reg class IA (p85-alpha), Vinculin, TGF-beta 3, IKK-alpha, TGF-beta receptor type II, Ski, MMP-2, EGR1, ACTA2, TGF-beta, Fyn, Tropomyosin-1, SNAIL1, AKT(PKB), Vimentin |
| 21 | Apoptosis and survival_Regulation of apoptosis by ESR1 and ESR2 | 3.03E-10 | PI3K reg class IA (p85), PI3K cat class IA, Protein kinase G1, RelA (p65 NF-kB subunit), Calcineurin A (catalytic), PI3K reg class IA (p85-alpha), PI3K reg class IA, ESR1 (nuclear), ERK1/2, Protein kinase G, CREB1, ESR1 (membrane), p90RSK2(RPS6KA3), NF-kB, Guanylate Cyclase 1, soluble, ERK2 (MAPK1), AKT(PKB), Caveolin-1, NF-kB p50/p65, MKK7 (MAP2K7), c-Src, PKA-cat (cAMP-dependent), MEK4(MAP2K4) |
| 22 | Development_The role of GDNF ligand family/ RET receptor in cell survival, growth and proliferation | 3.2E-10 | RAP-1A, p38alpha (MAPK14), ROCK, PI3K reg class IA (p85), PI3K cat class IA, B-Raf, GAB1, Cyclin A2, IRS-1, IKK-alpha, GFRalpha1, PSPN, CaMK II alpha, F-Actin cytoskeleton, ERK1/2, FAK1, CREB1, EGR1, p90RSK2(RPS6KA3), NF-kB, c-FLIP, FRS2, AKT(PKB), SHANK3, LIMK1, C3G, ARTN, c-Src, Paxillin, MEK4(MAP2K4) |
| 23 | Development_Role of HDAC and calcium/calmodulin-dependent kinase (CaMK) in control of skeletal myogenesis | 3.83E-10 | p38alpha (MAPK14), HDAC9, MEF2D, CARM1, PI3K cat class IA, Calcineurin A (catalytic), p38beta (MAPK11), MAP3K2 (MEKK2), PI3K reg class IA, NF-AT1(NFATC2), MAP3K3, IRS-1, MEF2C, 14-3-3, CACNA1C, MEF2A, HDAC7, MEF2, HDAC5, AKT(PKB), p300, NCOA2 (GRIP1/TIF2) |
| 24 | Development_VEGF signaling *via* VEGFR2 - generic cascades | 4.31E-10 | PI3K cat class IA, GSK3 beta, MAPKAPK2, Calcineurin A (catalytic), PKC, PI3K reg class IA, CCL2, Vinculin, PKC-alpha, HSP27, TCF7L2 (TCF4), ROCK1, ERK1/2, FAK1, CREB1, Actin cytoskeletal, Fyn, ERK2 (MAPK1), AKT(PKB), MNK1, NF-kB p50/p65, c-Src, TSAD, PLA2G5, p38 MAPK, ERK1 (MAPK3), NF-AT2(NFATC1), p90Rsk, MLCK, Paxillin |
| 25 | Immune response_IL-1 signaling | 5.12E-10 | KHSRP, p38alpha (MAPK14), JAM2, c-IAP2, PI3K reg class IA (p85), PI3K cat class IA, AP-1, RelA (p65 NF-kB subunit), MAPKAPK2, NF-kB1 (p105), NF-kB1 (p50), CD44, CCL2, MAP3K3, HSP27, TRAF6, ERK1/2, EGR1, FGF2, NF-kB, AKT(PKB), NIK(MAP3K14), IRF1, NF-kB p50/p65, MYLK1, PPAR-gamma, MEK4/7, MEK4(MAP2K4) |
| 26 | Signal transduction_Angiotensin II/AGTR1 signaling *via* Notch, Beta-catenin and NF-kB pathways | 5.39E-10 | NOTCH1 (NICD), CBP, GSK3 beta, RelA (p65 NF-kB subunit), NOTCH1 receptor, PKC, CCL2, IKK-alpha, TRAF6, TCF7L2 (TCF4), ROCK1, ERK1/2, MMP-2, ACTA2, SNAIL1, NF-kB, CTGF, ERK2 (MAPK1), AKT(PKB), NIK(MAP3K14), NF-kB p50/p65, p300, NOTCH1 (NEXT), ADAM17, p38 MAPK, PKA-cat (cAMP-dependent), YAP1 (YAp65) |
| 27 | Development_NOTCH/ TGF-beta crosstalk in EMT | 5.54E-10 | NOTCH1 (NICD), NOTCH2 (2ICD), NOTCH4 (ICD4), TGF-beta 2, TGF-beta 1, GSK3 beta, NOTCH1 receptor, HEYL, TGF-beta receptor type II, TCF7L2 (TCF4), SIP1 (ZFHX1B), Transgelin, NOTCH4 receptor, ACTA2, Calponin-1, SNAIL1, NOTCH2 receptor, Jagged1, NRF2, TCF8 |
| 28 | TGF-beta 1-induced transactivation of membrane receptors signaling in hepatocellular carcinoma (HCC) | 7.41E-10 | p130CAS, TGF-beta 1, PI3K cat class IA, GSK3 beta, ITGA2, PI3K reg class IA, PTEN, TGF-beta receptor type II, Lef-1, FAK1, c-Abl, PDGF-A, Cyclin A, TGF-beta, SNAIL1, AKT(PKB), PDGF receptor, LIMK1, c-Src, Actin, PDGF-R-beta |
| 29 | Immune response_Platelet activating factor/ PTAFR pathway signaling | 9E-10 | STAT2, PI3K cat class IA, MAPKAPK2, Calcineurin A (catalytic), PI3K reg class IA, NF-AT1(NFATC2), CCL2, Tyk2, IKK-alpha, Adenylate cyclase, F-Actin cytoskeleton, G-protein beta/gamma, NF-kB, AKT(PKB), NF-kB p50/p65, Arrestin 3, c-Src, p38 MAPK, NF-AT2(NFATC1), PKA-cat (cAMP-dependent), NF-AT, Beta-arrestin2 |
| 30 | HGF receptor (Met) and MSP receptor (RON) signaling in small cell lung cancer (SCLC) | 1.69E-09 | p38alpha (MAPK14), PI3K reg class IA (p85), PI3K cat class IA, MAPKAPK2, GAB1, PKR, ERK1/2, FAK1, PDGF-A, CREB1, CrkL, FGF2, Alpha adducin, AKT(PKB), Thrombospondin 1, HGF receptor (Met), c-Src, Paxillin, Gamma adducin |
| 31 | Apoptosis and survival_NGF/ TrkA PI3K-mediated signaling | 2.07E-09 | RAP-1A, MLCP (reg), ROCK, PI3K reg class IA (p85), TrkA, PI3K cat class IA, MRCKalpha, GSK3 beta, PARD3, ELMO2, Calcineurin A (catalytic), GAB1, Kalirin, MSN (moesin), N-WASP, MRLC, ERK1/2, CREB1, Actin cytoskeletal, VAV-3, AKT(PKB), LIMK1, c-Src, ARAP3, SSH1L, ILK |
| 32 | Development_Negative regulation of STK3/4 (Hippo) pathway and positive regulation of YAP/TAZ function | 2.17E-09 | MLCP (reg), TGF-beta 1, PARD3, Schwannomin (NF2), Nephrocystin-4, FRMD4A, STK4, CD44, STK3, EGFR, Itch, LARG, ERK1/2, ARHGEF1 (p115RhoGEF), LATS2, TAZ, G-protein alpha-q/11, S1P2 receptor, Actin, ERK1 (MAPK3), NEDD4, ILK, YAP1 (YAp65) |
| 33 | Glucocorticoid-induced elevation of intraocular pressure as glaucoma risk factor | 2.17E-09 | ROCK, PI3K cat class IA, WNT5A, PI3K reg class IA (p85-alpha), PI3K cat class IA (p110-beta), Elastin, EGFR, MMP-2, FAK1, Syndecan-4, Cdt6 (angiopoietin-like), Actin cytoskeletal, Alpha-actinin, Filamin A, CLIM1, Collagen IV, Thrombospondin 1, HGF receptor (Met), c-Src, PKC-epsilon, MLCK, CD47, FGFR1 |
| 34 | G-protein signaling_RhoA inhibition | 2.84E-09 | 14-3-3 beta/alpha, RhoGAP1, Rap1, ABL2, Protein kinase G1, MARK2, cAMP-GEFI, cAMP-GEFII, SMURF1, 14-3-3 zeta/delta, FAK1, ARHGEF1 (p115RhoGEF), Angiopoietin 1, TGF-beta, Fyn, FGF2, PRNP, Guanylate Cyclase 1, soluble, ERK2 (MAPK1), c-Src, PARD6, ARAP3, ERK1 (MAPK3), PKA-cat (cAMP-dependent), SLK, Myosin IXb |
| 35 | Signal transduction_Angiotensin II/ AGTR1 signaling *via* p38, ERK and PI3K | 3.05E-09 | ELAVL1 (HuR), p130CAS, PI3K cat class IA, RECK, PI3K reg class IA (p85-alpha), ALOX12, CCL2, KLF5, ETS1, EGFR, ERK1/2, MEF2C, MMP-2, PDGF-A, CREB1, EGR1, G-protein beta/gamma, MEF2A, Fyn, AKT(PKB), SP1, MNK1, c-Src, ADAM17, p38 MAPK, PKA-cat (cAMP-dependent), PLD2, p90Rsk, CaMK II delta, PDGF-R-beta |
| 36 | Development_Negative regulation of WNT/Beta-catenin signaling in the nucleus | 3.11E-09 | NF-AT5, KDM2, WNT5A, GSK3 beta, TRRAP, Calcineurin A (catalytic), WWOX, Nephrocystin-4, c-Cbl, BACH1, HBP1, Tcf(Lef), WNT, Lef-1, TCF7L2 (TCF4), ICAT, HIC1, 14-3-3, LATS2, RANBP3, GLI-3R, HIC5, CBP/P300, Dsh, PPAR-gamma, SOX9, TCF7 (TCF1), Frizzled |
| 37 | Cell adhesion_Integrin-mediated cell adhesion and migration | 3.12E-09 | Talin, p130CAS, PARD3, PKC, Vinculin, PINCH, Zyxin, 14-3-3 zeta/delta, F-Actin cytoskeleton, LARG, FAK1, ARHGEF1 (p115RhoGEF), Actin cytoskeletal, Alpha-parvin, Alpha-actinin, GIT2, Vinexin, VAV-3, Collagen IV, c-Src, PARD6, ILK, Paxillin |
| 38 | Immune response_Gastrin in inflammatory response | 3.13E-09 | ELAVL1 (HuR), p38alpha (MAPK14), MEF2D, PI3K reg class IA (p85), PI3K cat class IA, PKC-alpha, IRS-1, IKK-alpha, EGFR, TRAF6, LARG, MEF2C, FAK1, CREB1, MEF2, ERK2 (MAPK1), AKT(PKB), NIK(MAP3K14), G-protein alpha-q/11, NF-kB p50/p65, c-Src, PKC-epsilon, ERK1 (MAPK3), MEK4(MAP2K4) |
| 39 | Signal transduction_Endothelin-1/ EDNRA signaling | 3.5E-09 | MLCP (reg), p130CAS, Rap1, ROCK, PI3K reg class IA (p85), cPKC (conventional), PI3K cat class IA, PKC, Adenylate cyclase, SLC9A1, ERK1/2, MEF2C, FAK1, CREB1, ACTA2, G-protein beta/gamma, HDAC7, CTGF, HDAC5, Guanylate Cyclase 1, soluble, AKT(PKB), G-protein alpha-q/11, p300, c-Src, p38 MAPK, Ryanodine receptor 2, EDNRA |
| 40 | Development_MAG, Reticulon 4 and OMgp in inhibition of neurite outgrowth | 3.87E-09 | RAP-1A, MLCP (reg), SH3RF, NGFR (ICD), ROCK, NGFR(TNFRSF16), GSK3 beta, MARK2, NGFR (CTF), PTEN, Kalirin, EGFR, MRLC, LARG, FAK1, Syndecan-4, Actin cytoskeletal, HDAC6, AKT(PKB), TrkB, LIMK1, c-Src, S1P2 receptor, ADAM17, LRP1, Syndecan-3 |
| 41 | Signal transduction_Non-neuronal ACM1, ACM3 and ACM5 signaling | 4.18E-09 | MLCP (reg), ROCK, PI3K cat class IA, ITGA2, PKC, cAMP-GEFI, PI3K reg class IA, PKC-alpha, Adenylate cyclase, MRLC, ERK1/2, MEF2C, Protein kinase G, ARHGEF1 (p115RhoGEF), G-protein alpha-11, Guanylate Cyclase 1, soluble, AKT(PKB), G-protein alpha-q/11, SP1, ACM5, PLC-epsilon, PKA-cat (cAMP-dependent), PLD2, SRF, MLCK |
| 42 | Development_Canonical TGF-beta signaling | 4.38E-09 | NOTCH1 (NICD), TGF-beta 2, CBP, TGF-beta 1, AP-1, Miz-1, E2A, TGF-beta 3, SMAD7, ETS1, TGF-beta receptor type II, Ski, Lef-1, Itch, SIP1 (ZFHX1B), CDH1, TGF-beta, SNAIL1, SP1, p300, Jagged1, Vimentin, TCF8, ILK |
| 43 | Development_Positive regulation of WNT/Beta-catenin signaling in the nucleus | 4.38E-09 | Casein kinase II, alpha chains, Kindlin-2, CBP, GSK3 beta, Tcf(Lef), RUNX, WNT, Lef-1, beta-TrCP, TCF7L2 (TCF4), FOXP1, ICAT, ERK2 (MAPK1), p300, CBP/P300, LRRFIP2, NCOA2 (GRIP1/TIF2), Dsh, SOX9, TCF7 (TCF1), Frizzled, UBR5, JRK, YAP1 (YAp65) |
| 44 | Immune response_Function of MEF2 in T lymphocytes | 5.25E-09 | p38alpha (MAPK14), HDAC9, MEF2D, CARM1, Calcineurin A (catalytic), p38beta (MAPK11), MAP3K2 (MEKK2), NF-AT1(NFATC2), MAP3K3, MEF2C, 14-3-3, CACNA1C, CABIN1, MEF2A, HDAC7, MEF2, HDAC5, p300, LAT, NCOA2 (GRIP1/TIF2) |
| 45 | Protein folding and maturation_Regulation of amyloid precursor protein processing | 5.47E-09 | GGA2, X11, Rap1, NGFR(TNFRSF16), TrkA, GSK3 beta, betaAPPs, cAMP-GEFI, alphaAPPs, BACE1, GBR1, Adenylate cyclase, Tiam2, Calsyntenin-1, F-spondin, APP-C99, G-protein beta/gamma, Amyloid beta 40, PICALM, GGA3, APP, DAB2, KLC1, Amyloid beta, SFK, c-Src, ADAM17, APP-C59 (AICD), LRP1, Amyloid beta 42, RAP6 |
| 46 | Deficient alpha-MSH signaling in melanoma | 6.24E-09 | p38alpha (MAPK14), SOX10, PI3K cat class IA, GSK3 beta, B-Raf, PI3K reg class IA, PKA-cat alpha, ROCK1, ERK1/2, CREB1, TYRP2, AKT(PKB), p38 MAPK, MITF, PKA-cat (cAMP-dependent), SOX9 |
| 47 | Ovarian cancer (main signaling cascades) | 6.29E-09 | PI3K cat class IA (p110-alpha), PI3K cat class IA, GSK3 beta, B-Raf, PI3K reg class IA, ESR1 (nuclear), PTEN, IKK-alpha, Tcf(Lef), EGFR, ERK1/2, MMP-2, CREB1, G-protein beta/gamma, SNAIL1, NF-kB, AKT(PKB), SP1, HGF receptor (Met), c-Src, PKA-cat (cAMP-dependent), ILK, EDNRA |
| 48 | Role of Tissue factor-induced Thrombin signaling in cancer | 6.29E-09 | MLCP (reg), ROCK, PI3K cat class IA (p110-alpha), RelA (p65 NF-kB subunit), PI3K reg class IA (p85-alpha), PKC-alpha, IKK-alpha, EGFR, MRLC, LARG, ERK1/2, MMP-2, FAK1, Angiopoietin 1, Actin cytoskeletal, ERK2 (MAPK1), AKT(PKB), G-protein alpha-q/11, NF-kB p50/p65, c-Src, ERK1 (MAPK3), MLCK, Paxillin |
| 49 | Cell adhesion_PLAU signaling | 6.29E-09 | Casein kinase II, alpha chains, p130CAS, c-IAP2, ROCK, PI3K cat class IA, PI3K reg class IA (p85-alpha), JAK1, Tyk2, IKK-alpha, c-IAP1, EGFR, F-Actin cytoskeleton, MRLC, ERK1/2, FAK1, NF-kB, AKT(PKB), Caveolin-1, MYLK1, c-Src, MLCK, Paxillin, PDGF-R-beta |
| 50 | Immune response_CTLA-4 signaling | 8.82E-09 | RAP-1A, PI3K reg class IA (p85), CD80, PI3K cat class IA, AP-1, PKC-eta, NF-AT1(NFATC2), FOXP3, Itch, ERK1/2, CBL-B, Fyn, GIT2, NF-kB, ERK2 (MAPK1), AKT(PKB), NF-kB p50/p65, C3G, ALPHA-PIX, ERK1 (MAPK3), NF-AT2(NFATC1), MLCK, NF-AT |
